# Alkali injury–induced pathological lymphangiogenesis in the iris facilitates the infiltration of T cells and ocular inflammation

**DOI:** 10.1172/jci.insight.175479

**Published:** 2024-04-08

**Authors:** Zheng Liu, Keli Liu, Shunhua Shi, Xun Chen, Xinyu Gu, Weifa Wang, Keli Mao, Rukeye Yibulayi, Wanwen Wu, Lei Zeng, Weibin Zhou, Xiaofeng Lin, Feng Zhang, Bingsheng Lou

**Affiliations:** 1 State Key Laboratory of Ophthalmology, Zhongshan Ophthalmic Center, Sun Yat-sen University, Guangdong Provincial Key Laboratory of Ophthalmology and Visual Science, Guangzhou, China.; 2 Sun Yat-sen Memorial Hospital, Sun Yat-sen University, Guangzhou, China.

**Keywords:** Immunology, Ophthalmology, Lymph, Retinopathy, T cells

## Abstract

Inflammatory lymphangiogenesis is intimately linked to immune regulation and tissue homeostasis. However, current evidence has suggested that classic lymphatic vessels are physiologically absent in intraocular structures. Here, we show that neolymphatic vessels were induced in the iris after corneal alkali injury (CAI) in a VEGFR3-dependent manner. *Cre-loxP*–based lineage tracing revealed that these lymphatic endothelial cells (LECs) originate from existing Prox1^+^ lymphatic vessels. Notably, the ablation of iridial lymphangiogenesis via conditional deletion of *VEGFR3* alleviated the ocular inflammatory response and pathological T cell infiltration. Our findings demonstrate that iridial neolymphatics actively participate in pathological immune responses following injury and suggest intraocular lymphangiogenesis as a valuable therapeutic target for the treatment of ocular inflammation.

## Abstract 2

Iridial neo-lymphatics actively participate in pathological immune responses following injury, supporting intraocular lymphangiogenesis as a potential therapeutic target for the treatment of ocular inflammation.

## Introduction

The lymphatic vasculature is a unidirectional conduit system developed in most organs and tissues that is crucial for fluid hemostasis, immune defense, and intestinal lipid absorption (1). Under pathological circumstances, inflammatory cues or tumors can trigger the growth or expansion of regional lymphatic vessels within adult tissues in a process called pathological lymphangiogenesis, which is considered a viable therapeutic target for inflammatory disease (2–7).

Upon initiation of inflammation, inflammatory cytokines such as TNF and IL-1 are released to recruit and activate immune cells, which further produce a class of chemokines/cytokines to induce lymphangiogenesis in inflamed peripheral tissue (8–10). It has been recognized that macrophages are the most critical immune cells in driving this process, and they act via secretion of lymphangiogenic VEGFC, VEGFD, and VEGFA to induce local sprouting and remodeling of preexisting lymphatic endothelial cells (LECs), primarily through the engagement of the VEGFC/VEGFR3 signaling pathway (10–13). Interestingly, some CD11b^+^ macrophages express VEGFR3 and have been reported to transdifferentiate into LECs under inflammatory conditions (14–16), although more mechanistic insights into the cellular origin of inflammation-associated lymphangiogenesis are needed.

During acute inflammation, the lymphatic network undergoes expansion to fulfill the increased demand for fluid and antigen clearance and leukocyte trafficking; it then regresses following the resolution of inflammation (17). Recent studies have shown that improvement of cardiac lymphatic growth and function could promote myocardial infarction recovery and delay atherosclerotic plaque formation (18–20). Likewise, meningeal lymphatics and the glymphatic system can be exploited to facilitate the removal of β-amyloid or other toxic remnants in neurodegenerative diseases (21, 22). However, the ablation of meningeal lymphatics can relieve the pathology of experimental autoimmune encephalomyelitis, which is used as a model to mimic autoreactive T cell attack in multiple sclerosis (5, 23). Accumulating evidence also suggests that lymphangiogenesis promotes antigen presentation to the recipient’s lymph node and provokes adverse immune responses in graft rejection (24–26). In allergic diseases, the detrimental effect of lymphangiogenesis has also been recognized (27, 28). Lymphangiogenesis has context-dependent roles in the resolution of chronic inflammation.

The eye is one of a few organs in the body with immune privilege, and intraocular tissues, including the retina, choroid, lens, ciliary body, and iris, are considered to be physiologically devoid of classic lymphatic vessels (29–32). To maintain ocular immune privilege, endogenous inhibitors such as vasoactive intestinal peptide and α-melanocyte–stimulating hormone are secreted in the aqueous humor and inhibit lymphangiogenesis (33). Likewise, the cornea is also well recognized as a lymphatic-free tissue due to the presence of endogenously antilymphangiogenic factors such as TSP-1 and ectopic VEGFR3 decoy in its local microenvironment (34). However, the alymphatic nature of the cornea can be disrupted by severe injury or infection (14, 35). For example, corneal alkali injury (CAI) can effectively trigger corneal lymphangiogenesis (35, 36). Alkali burns immediately cause damage, cause loss of corneal and limbal epithelial and stromal cells, and result in immune cell activation and the production of proinflammatory cytokines that act to induce neolymphatic outgrowth into the cornea from the existing lymphatic plexus in the limbus (37, 38). Penetration of alkali also promptly elevates the pH in the aqueous chamber and damages the adjacent iridial tissue, leading to severe intraocular inflammation and complications (36, 39–41). This is often associated with evident angiogenesis in the iris (36, 39), but whether lymphangiogenesis occurs in the injured iris or other intraocular tissues remains to be determined.

Here, we show that CAI also triggers lymphangiogenesis in the iris that is free of lymphatic coverage under healthy conditions. *Cre-loxP*–based EC and LEC lineage tracing revealed that CAI-induced iridial neolymphatics originated from existing lymphatics. Via VEGFC/VEGFR3 loss-of-function and gain-of-function approaches, we found that iridial lymphangiogenesis led to exacerbated regional inflammation and retinal injury, indicating that it intimately regulates intraocular inflammation. Our results reveal the presence of intraocular neolymphatics in ocular inflammation.

## Results

### Lymphangiogenesis in the iris after CAI.

**Figure 1:**
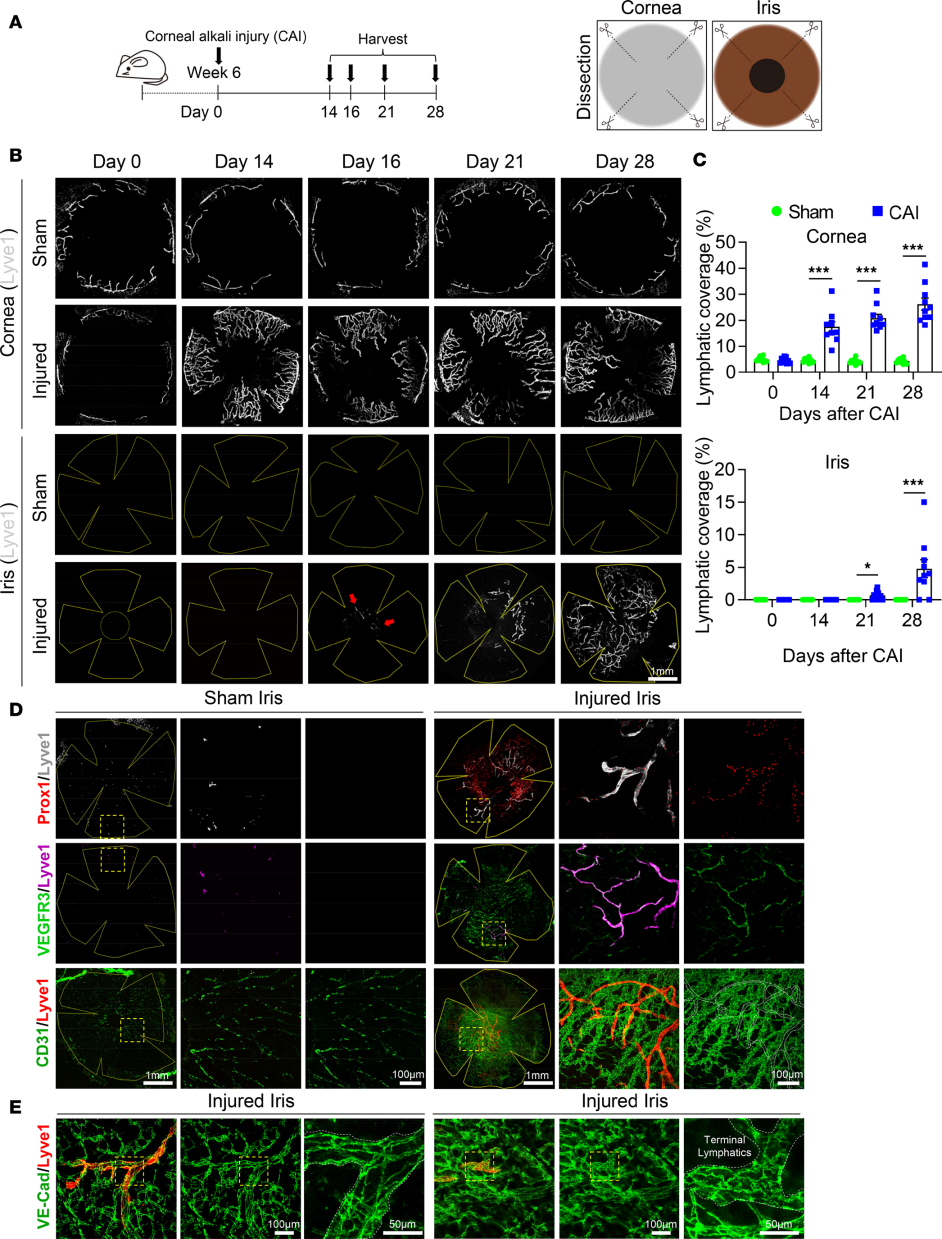
Corneal alkali injury (CAI) induces iridial lymphangiogenesis. (**A**) Schematic showing experimental timeline of CAI and analysis of lymphangiogenesis in the cornea and iris on days 0, 14, 16, 21, and 28. (**B**) Immunofluorescence staining of LYVE1^+^ lymphatic vessels in cornea and iris tissue whole mounts from mice at the indicated days after CAI or sham treatment. The CAI or sham treatment was done by placing a 2 mm round filter paper infiltrated with 0.5 μL of 1 M sodium hydroxide solution (CAI) or PBS (sham) on the cornea for 60 seconds. Note that sparse LYVE1^+^ neolymphatics initially formed in the iris whole mounts on day 16 after CAI and underwent expansion thereafter (see arrows). Scale bar: 1 mm. (**C**) Quantitation of Lyve1^+^ lymphatic covered area in the cornea and iris from mice received CAI or sham treatment at the indicated time points following CAI. All data are mean ± SEM. *n* = 10 mice per group. Each dot represents 1 mouse. *P < 0.05, **P < 0.01, ***P < 0.001. Mann-Whitney *U* test. (**D**) Characterization of expression of common lymphatic endothelial cell (LEC) markers (LYVE1, Prox1, VEGFR3, and CD31) in CAI-induced iridial neolymphatics in mice, compared with sham control mice, on day 28 after the treatment. Scale bars: 1 mm and 100 μm. (**E**) VE-cadherin and Lyve1 staining of the iris tissue whole mounts from mice on day 28 after the CAI or sham treatment. Scale bars: 100 μm and 50 μm.

Using the well-established CAI mouse model, we determined that injury can disrupt the alymphatic nature of the iris. Sparse LYVE1^+^ neolymphatics were initially detected in the iris whole mounts starting at day 16 after CAI (Figure 1, A and B). From days 21 through 28, the injured iris had marked expansion of neolymphatics (Figure 1, B and C). This was accompanied by drastic vascular remodeling characterized by significant neovascularization and collagen deposition in the anterior segment (Supplemental Figure 1, A and B; supplemental material available online with this article; https://doi.org/10.1172/jci.insight.175479DS1). In comparison, corneal lymphangiogenesis occurred much earlier than iridial lymphangiogenesis, as more pronounced LYVE1^+^ lymphatic sprouts outside of the limbal vascular plexus were readily observed in corneal whole mounts from days 14 through 28 following CAI (Figure 1, A–C). By contrast, the sham control iris and cornea remained alymphatic (Figure 1, B and C). These iridial neolymphatics were morphologically different from corneal lymphatics, characterized by more branches and narrower vessel diameters (Supplemental Figure 1, C and D). No significant differences between male and female mice were observed in CAI-induced iridial and corneal lymphangiogenesis (vessel coverage, width and branches; Supplemental Figure 1E).

Next, we characterized the cellular features of the iridial neolymphatics conventional LEC and EC markers. Expression of Prox1 and VEGFR3, master lymphatic regulators essential in LEC specification and proliferation (42, 43), was observed in line with LYVE1^+^ staining in the neolymphatics on day 28 after CAI (Figure 1, D and E), confirming their lymphatic features. Moreover, the panendothelial markers VE-cadherin and CD31 were expressed in these LYVE1^+^ lymphatic vessels and the surrounding blood vessels (Figure 1, D and E). Notably, VE-cadherin staining also revealed that, on day 28 after CAI, LECs in the terminal segment of iridial neolymphatic vessels were adjoined by both button and zipper-like junctions, whereas the following lymphatic plexus contained primarily zipper-like junctions (Figure 1E), indicating that the neolymphatics were functional (44). Collectively, these data suggest that CAI induces iridial lymphangiogenesis.

### CAI-induced iridial neolymphatics originate from existing lymphatic vessels.

**Figure 2:**
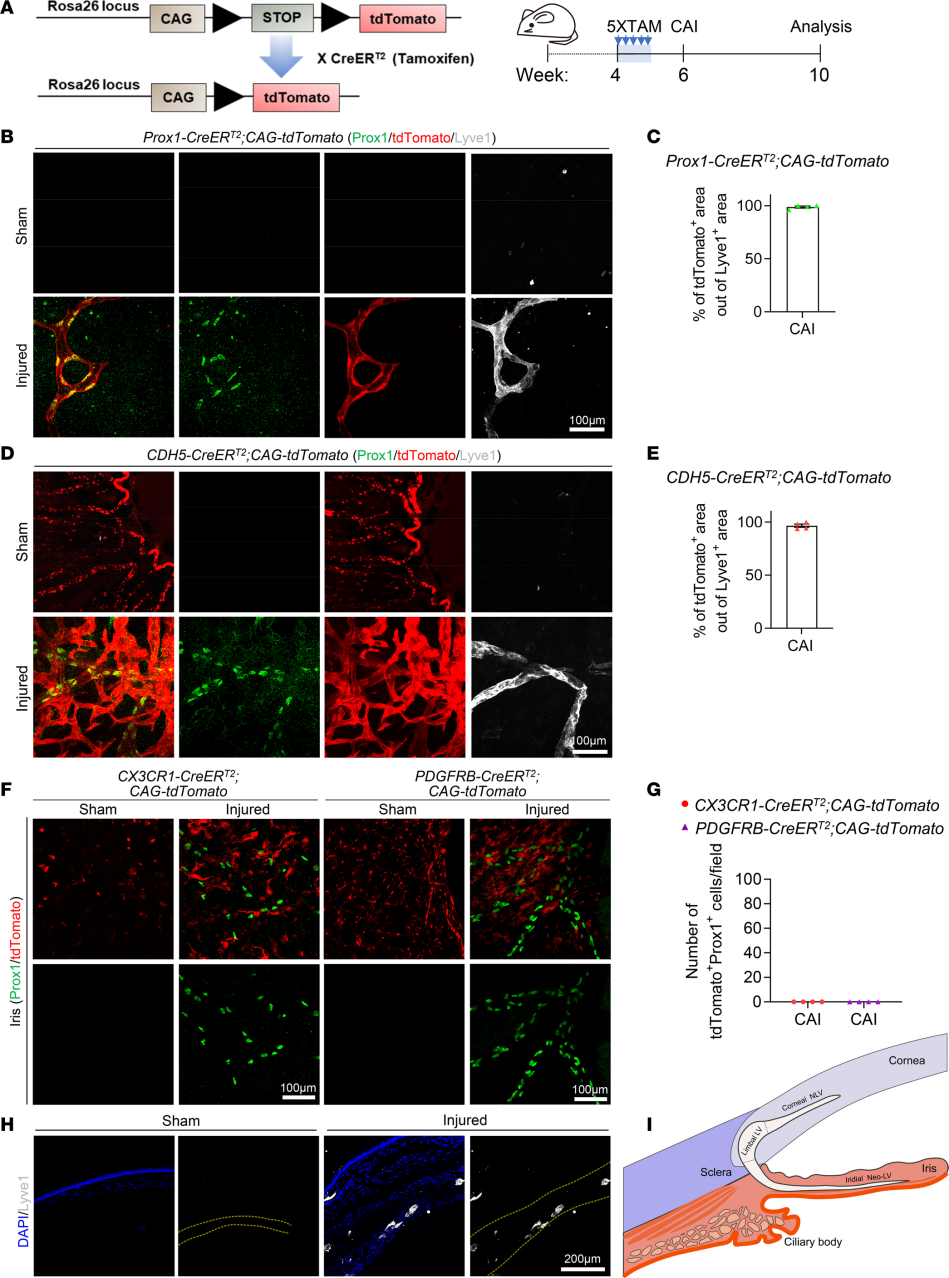
LECs in CAI-induced neolymphatics have a Prox1^+^, CDH5^+^, PDGFRβ^–^, and CX3CR1^–^ lineage. (**A**) Schematic showing generation of mice harboring the *CAG-tdTomato* reporter and *CreER^T2^* transgene and experimental timeline of tamoxifen (TAM) administration and CAI for lineage tracing in the indicated mouse lines in **B**–**G**. Arrows indicate TAM administered 5 times (80 mg/kg) i.p. injections in week 4, followed by CAI treatment at week 6 and analysis at week 10. (**B** and **D**) Representative tdTomato, Prox1, and Lyve1 immunostaining images of the iris whole mounts from *Prox1-CreER^T2^;CAG-tdTomato* (**B**) or *CDH5-CreER^T2^;CAG-tdTomato* (**D**) mice at week 10 that received TAM administered 5 times i.p. in week 4 and CAI treatment at week 6. Note overlapping of tdTomato-labeled area with Prox1/Lyve1-labeled lymphatic areas in both mouse lines with CAI treatment, indicating that LECs in CAI-induced neolymphatics originate from Prox1^+^ and CDH5^+^ existing lymphatics. Scale bars: 100 μm. (**C** and **E**) Quantification of the ratio of tdTomato-labeled area and Prox1/Lyve1-labeled area in **B** and **D**. Data are shown as mean ± SEM. *n* = 4 mice per group. Each dot represents 1 mouse. (**F**) Representative immunostaining images of tdTomato and Prox1 of the iris whole mounts from *PDGFRB-CreER^T2^;CAG-tdTomato* or *CX3CR1-CreER^T2^;CAG-tdTomato* mice at week 10 that received TAM administered 5 times i.p. in week 4 and CAI or sham treatment at week 6. Note that Prox1 staining does not overlap with tdTomato-labeled area in both mouse lines with CAI treatment, indicating that LECs in CAI-induced neolymphatics do not originate from PDGFRβ^+^ mural cells or CX3CR1^+^ myeloid cells. Scale bar: 100 μm. (**G**) Quantification of number of Prox1 and tdTomato double-positive cells in **F**. Data are shown as mean ± SEM. *n* = 4 mice per group. Each dot represents 1 mouse. (**H**) Immunofluorescence staining for Lyve1 in sagittal cryosections of the anterior segment of the eyes from WT mice at week 10 that received CAI or sham treatment at week 6. The dotted line depicts the injured iris. Scale bar: 200 μm. (**I**) Diagram showing possible route of the induction of the iridial neolymphatics from existing limbal lymphatic vessels (LVs).

Previous lineage-tracing studies suggested that neolymphatics can be derived from existing lymphatic vascular trees (45) or transdifferentiated from other cells of venous (46, 47) or nonvenous (14, 48) origins. To determine the origin of CAI-induced iridial neolymphatics, we performed genetic cell fate tracking experiments using *CAG-tdTomato* reporter mice (49) that were intercrossed with various tamoxifen-inducible (TAM-inducible) *Cre* lines (Figure 2A). TAM was administered to the respective reporter mice at week 4, and immunofluorescence studies of the limbal vascular plexus from mice at week 6 revealed that nearly all existing LECs counterstained by Prox1 were genetically labeled by *tdTomato* driven by *Prox1-CreER^T2^* (50) or *CDH5-CreER^T2^* (51), despite varying tdTomato expression intensities in LECs versus blood ECs (Supplemental Figure 2, A–C). These results validated the high efficiency and specificity of genetic labeling of existing LECs using the *Prox1-CreER^T2^*;*CAG-tdTomato* or *CDH5-CreER^T2^;CAG-tdTomato* approach. In contrast, tdTomato fluorescence was completely absent in Prox1^+^ limbal LECs in *PDGFRB-CreER^T2^;CAG-tdTomato*; (52) or *CX3CR1-CreER^T2^*;*CAG-tdTomato* (53) reporter mice. We then subjected these reporter mice to the CAI or sham treatment at week 6 and probed tdTomato expression in the iris at week 10 (Figure 2A). Our data show that LYVE1^+^ neolymphatics in the injured iris were predominantly labeled by Prox1-tdTomato and CDH5-tdTomato (Figure 2, B–E), suggesting that iridial neolymphatics were primarily derived from existing CDH5^+^Prox1^+^ LECs. To investigate the cell origin of CAI-induced iridial neolymphatics more rigorously, we also analyzed *PDGFRB-CreER^T2^*;*CAG-tdTomato* and *CX3CR1-CreER^T2^;CAG-tdTomato* mice (Supplemental Figure 2, D and E). However, we did not observe that PDGFRβ-tdTomato or CX3CR1-tdTomato fluorescence colocalized with the counterstained Prox1 (Figure 2, F and G), indicating that CAI-induced iridial neolymphatics were unlikely to be a result of de novo lymphangiogenesis via transdifferentiating of mural cells (48) or macrophages (14, 15). Since immunostaining of the sagittal cryosections from the injured anterior chamber failed to identify any physical connections of the iridial lymphatics with the cornea (Figure 2H) and the iris directly connects with the limbus that possesses lymphatic vasculature, we concluded that CAI-induced iridial neolymphatics originated from existing lymphatics in, presumably, the limbus (Figure 2I).

### CAI-induced iridial lymphangiogenesis is mediated by VEGFC/VEGFR3 signaling.

**Figure 3:**
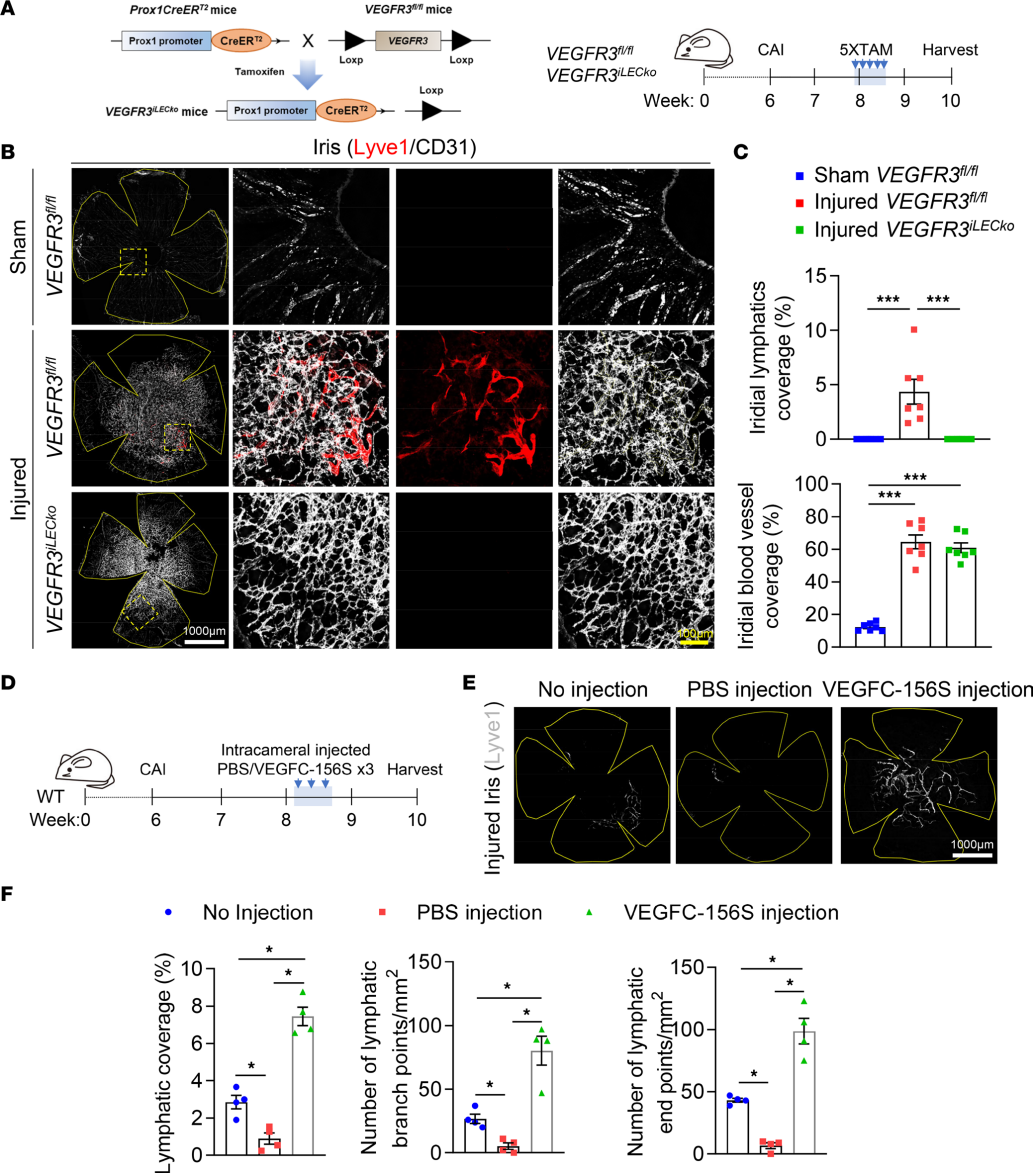
CAI-induced iridial lymphangiogenesis depends on VEGFC/VEGFR3 signaling. (**A**) Schematic showing generation of mice harboring the *VEGFR3^fl/fl^* allele and *Prox1*-*CreER^T2^* transgene and experimental timeline of TAM administration for conditional deletion of the *VEGFR3* gene (referred to as *VEGFR3^iLECko^*) and CAI in **B** and **C**. Mice received CAI or sham treatment at week 6, followed by TAM administered 5 times (80 mg/kg) i.p. between days 13 and 17 and analysis on day 28 after CAI. (**B**) CD31 and Lyve1 immunostaining images of the iris whole mounts from *VEGFR3^iLECko^* and *VEGFR3^fl/fl^* mice that received CAI or sham treatment at week 6, followed by TAM administered 5 times (80 mg/kg) i.p. between days 13 and 17 and analysis on day 28 after CAI. Note ablation of lymphatics in the injured *VEGFR3^iLECko^* mouse group. Scale bars: 1,000 μm and 100 μm. (**C**) Quantification of Lyve1^+^ lymphatic vessel and CD31^+^ blood vessel coverage in the iris in mice in **B**. Data are shown as mean ± SEM. *n* = 7 mice per group. Each dot represents 1 mouse. ***P < 0.001. One-way ANOVA with the Tukey post hoc test. (**D**) Experimental timeline of VEGFC-156S administration and CAI in **E** and **F**. Mice received CAI or sham treatment at week 6, followed by 150 ng administered 3 times VEGFC-156S or PBS intracameral injections between days 16 and 18 and analysis on day 28 after CAI. (**E**) Lyve1 immunostaining images of the iris whole mounts from WT mice that received CAI treatment at week 6, followed by 150 ng administered 3 times VEGFC-156S or PBS intracameral injections and analysis on day 28 after CAI. Note that intracameral injections with PBS alone could inhibit CAI-induced iridial lymphangiogenesis, compared with no injection controls, and that VEGFC-156S administration could promote CAI-induced iridial lymphangiogenesis. Scale bar: 1,000 μm. (**F**) Quantification of Lyve1^+^ lymphatic vessel coverage, branches, and terminal endpoints in the iris in mice in **E**. Data are shown as mean ± SEM. *n* = 4 mice per group. Each dot represents 1 mouse. *P < 0.05. Mann-Whitney *U* test.

We next investigated the involvement of the VEGFC/VEGFR3 pathway, the primary lymphangiogenic signaling pathway (17), in iridial lymphangiogenesis following CAI. We previously established inducible LEC-specific deletion of *VEGFR3* using the *VEGFR3^fl/fl^;Prox1-CreER^T2^* (referred to as *VEGFR3^iLECko^*) mouse line (27). Since iridial lymphatics were initially detected at approximately day 16 after CAI, we chose to induce *VEGFR3* deletion on days 13–17 via daily i.p. injections of TAM (Figure 3A). Whole-mount immunostaining of the iris using LYVE1 and CD31 antibodies revealed that CAI-induced iridial lymphangiogenesis was specifically blocked in *VEGFR3^iLECko^* mice, while neovascularization in the iris remained unaltered upon LEC-specific deletion of *VEGFR3* (Figure 3, B and C). Similarly, *VEGFR3* ablation resulted in largely inhibited corneal lymphangiogenesis following CAI (Supplemental Figure 3, A and B). Iridial and corneal lymphangiogenesis were inhibited in male and female *VEGFR3^iLECko^* mice to similar extents, reinforcing the notion that sex does not influence injury-induced ocular lymphangiogenesis (Supplemental Figure 3C).

In a complementary approach, we employed recombinant VEGFC-Cys156Ser (VEGFC-156S), which exclusively binds to VEGFR3 and selectively induces lymphangiogenesis without an apparent effect on angiogenesis (54). Interestingly, we found that intracameral injections of PBS alone led to significantly reduced CAI-induced iridial lymphangiogenesis compared with the noninjection controls (Figure 3, D–F). Nevertheless, 3 intracameral injections of 150 ng VEGFC-156S between days 16 and 18 after CAI significantly promoted the formation of iridial neolymphatics, as suggested by the increased coverage, branches, and terminal endpoints of neolymphatic vessels compared with the PBS injection group (Figure 3, D–F). Taken together, these results demonstrate the requirement of VEGFC/VEGFR3 signal for CAI-induced iridial lymphangiogenesis.

### Involvement of iridial neolymphatics in ocular inflammation following CAI.

**Figure 4:**
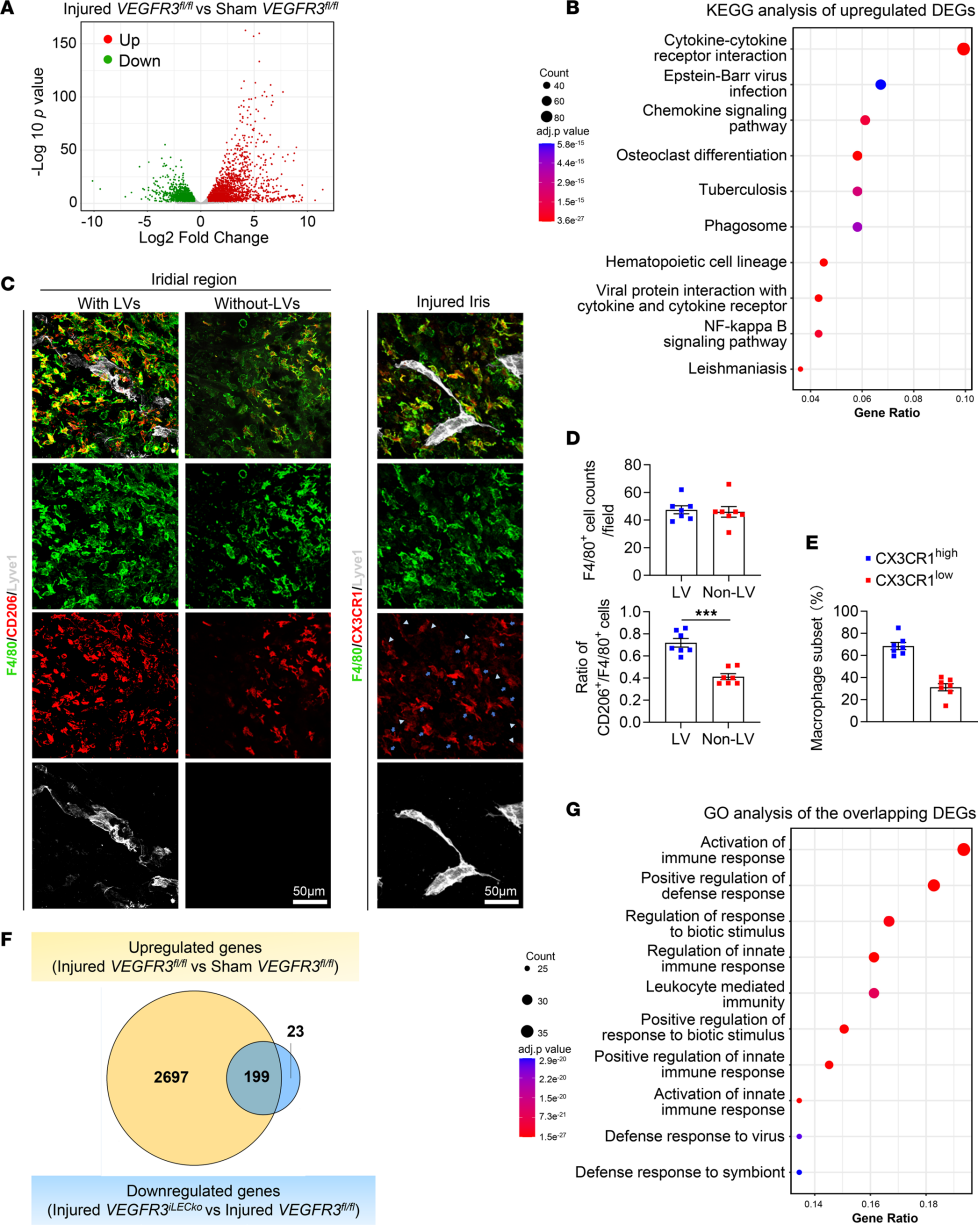
Transcriptomic characterization of CAI-treated iris tissues. (**A**) Volcano plot showing CAI-induced 2,896 upregulated differentially expressed genes (DEGs) and 2,332 downregulated DEGs in the iris, as revealed by bulk RNA-Seq analysis of iris tissues dissected from *VEGFR3^fl/fl^* mice with CAI or sham treatment. (**B**) KEGG analysis of upregulated DEGs in **A**. Dot size and color scale intensities represent counts of DEGs and *P*_adj_ values of the enriched pathways, respectively. (**C**) Immunostaining images for F4/80, CD206, and CX3CR1 in the iridial tissue regions with or without lymphatic vessel (LV) coverage in CAI-treated *VEGFR3^fl/fl^* mice. Arrows and arrowheads indicate CX3CR1^hi^ and CX3CR1^lo^ macrophages, respectively. Scale bar: 50 μm. (**D**) Quantification of CD206^+^ (M2) macrophage counts out of F4/80^+^ total macrophage counts in **C** (*n* = 7). Data are shown as mean ± SEM. *n* = 7 mice per group. Each dot represents 1 mouse. ***P < 0.001. Mann-Whitney *U* test. (**E**) Quantification of CX3CR1^hi^ and CX3CR1^lo^ subsets in F4/80^+^ total macrophage population in **C**. Data are shown as mean ± SEM. *n* = 7 mice per group. Each dot represents 1 mouse. (**F**) Venn diagram showing intersection between the upregulated DEGs in the injured *VEGFR3^fl/fl^* versus Sham *VEGFR3^fl/fl^* mouse groups and the downregulated DEGs in the injured *VEGFR3^iLECko^* versus injured *VEGFR3^fl/fl^* mouse groups. (**G**) GO analysis of the overlapping 199 DEGs in **F**. Dot size and color scale intensities represent counts of DEGs and *P*_adj_ values of the enriched pathways, respectively.

To evaluate the pathological effect of iridial neolymphatics on ocular inflammation, we performed RNA-Seq analysis using the iris tissues from PBS-treated Sham *VEGFR3^fl/fl^* mice, CAI-treated injured *VEGFR3^fl/fl^* mice, and CAI-treated injured *VEGFR3^iLECko^* mice. Differentially expressed gene (DEG) (>1.5-fold change, adjusted P value (*P*_adj_ < 0.05) analysis of the RNA-Seq data revealed a total of 2,896 upregulated and 2,332 downregulated genes in the injured *VEGFR3^fl/fl^* versus Sham *VEGFR3^fl/fl^* iris tissues (Figure 4A). Kyoto Encyclopedia of Genes and Genomes (KEGG) analysis and gene set enrichment analysis (GSEA) revealed that these upregulated DEGs were linked to the activation of pathways responsible for acute inflammatory and immune responses, such as cytokine receptor interactions and the NF-κB signaling pathway, confirming the drastic damage to intraocular tissues following CAI (Figure 4B and Supplemental Figure 4A). Consistent with this, massive accumulation of F4/80^+^ macrophages was observed within the interstitial space and around neolymphatics in the injured iris (Figure 4, C–E). These macrophages had both CX3CR1^hi^ and CX3CR1^lo^ populations (Figure 4, C and E), indicating the accumulation of both residential and infiltrating macrophages (55) in the injured iris. The vast majority of these macrophages are CD206^+^ cells (Figure 4, C and D), implying a M2 polarized phenotype (56).

In the injured *VEGFR3^iLECko^* versus injured *VEGFR3^fl/fl^* cohort, DEG analysis revealed 259 upregulated and 222 downregulated genes (Supplemental Figure 4B). We then performed a Venn diagram analysis between the downregulated DEGs in the injured *VEGFR3^iLECko^* versus injured *VEGFR3^fl/fl^* cohort and the upregulated DEGs in the injured *VEGFR3^fl/fl^* versus Sham *VEGFR3^fl/fl^* cohort to identify genes of interest that were involved in regulating iridial neolymphatics-mediated inflammatory responses (Figure 4F). Gene Ontology (GO) analysis of the 199 overlapping DEGs confirmed highly enriched gene sets involved in the regulation of innate and defense immune responses and leukocyte-mediated immunity (Figure 4G).

**Figure 5:**
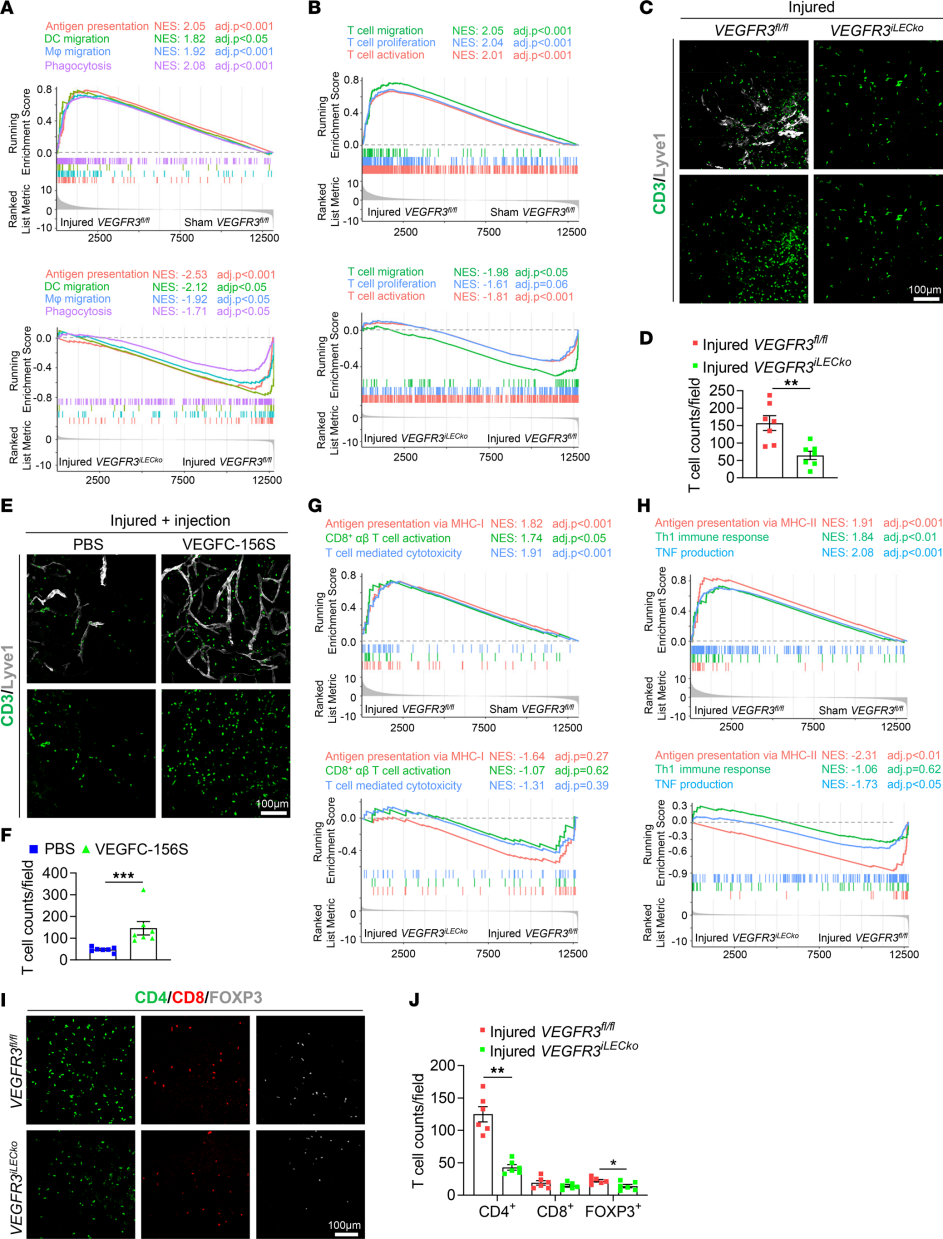
Iridial neolymphatics promote phagocytosis, antigen presentation, and T cell infiltration/activation following CAI. (**A** and **B**) GSEA showing expression profiles of phagocytosis/antigen presentation/DC/macrophage/T cell–related immunogenic pathways in the iris tissues following CAI and *VEGFR3* depletion. *P*_adj_ < 0.05 (after Benjamini-Hochberg multiple testing corrections) is considered statistically significant. NES, normalized enrichment score. (**C**) CD3 and Lyve1 staining of the iris tissue whole mounts from *VEGFR3^iLECko^* and *VEGFR3^fl/fl^* mice that received CAI at week 6, followed by TAM administered 5 times i.p. between days 13 and 17 and analysis on day 28 after CAI. (**D**) Quantification of CD3^+^ T cell counts (per 20× field) in **C**. Data are shown as mean ± SEM. *n* = 7 mice per group. Each dot represents 1 mouse. **P < 0.01. Mann-Whitney *U* test. (**E**) CD3 and Lyve1 staining of the iris tissue whole mounts from WT mice that received CAI at week 6, followed by VEGFC-156S or PBS intracameral injections administered 3 times between days 16 and 18 and analysis on day 28 after CAI. Note that intracameral injections with PBS alone could alleviate T cell infiltration to the injured iris and that VEGFC-156S administration could promote T cell infiltration following. (**F**) Quantification of CD3^+^ T cell counts (per 20× field) in **E**. Data are shown as mean ± SEM. *n* = 7 mice per group. Each dot represents 1 mouse. ***P < 0.001. Mann-Whitney *U* test. (**G** and **H**) GSEA showing expression profiles of the indicated antigen presentation/T cell–related immunogenic pathways in the iris tissues following CAI and *VEGFR3* depletion. *P*_adj_ < 0.05 (after Benjamini-Hochberg multiple testing corrections) is considered statistically significant. NES, normalized enrichment score. (**I**) CD4, CD8, and FOXP3 staining of the iris tissues from *VEGFR3^iLECko^* and *VEGFR3^fl/fl^* mice that received CAI at week 6, followed by TAM administered 5 times i.p. between days 13 and 17 and analysis on day 28 after CAI. (**J**) Quantification of CD4^+^, CD8^+^, and FOXP3^+^ T cell counts (per 20× field) in **I**. Scale bars: 100 μm (**C**, **E**, and **I**). Data are shown as mean ± SEM. *n* = 6 mice per group. Each dot represents 1 mouse. *P < 0.05, **P < 0.01. Mann-Whitney *U* test.

To investigate the immunoregulatory roles of neolymphatics in the injured iris, we performed GSEA for the inflammatory pathways caused by tissue injury. As expected, alkali injury led to evidently upregulated gene sets responsible for macrophage/DC migration, phagocytosis, and antigen processing/presentation (Figure 5A, upper panel), while these pathways were significantly suppressed by *VEGFR3* deletion (Figure 5A, lower panel). Furthermore, T cell migration, activation, and proliferation pathways were drastically induced in the iris following CAI (Figure 5B, upper panel). Most of these pathways were downregulated in the injured *VEGFR3^iLECko^* mouse group, compared with the injured *VEGFR3^fl/fl^* group (Figure 5B, lower panel). Whole-mount staining of the injured iris confirmed that *VEGFR3* deletion resulted in significantly decreased CD3^+^ T cell counts (Figure 5, C and D). Conversely, intraocular administration of VEGFC-156S led to increased CD3^+^ T cell infiltration in the injured iris (Figure 5, E and F).

GSEA further revealed that CAI induced evident activation of antigen presentation via MHC-I and -II and CD8^+^, CD4^+^, and Treg-mediated pathways in the iris (Figure 5, G and H, and Supplemental Figure 5C, left panel). The ablation of iridial neolymphatics via *VEGFR3^iLECko^* appeared to inhibit CD4^+^ T cell–mediated immune responses, as suggested by the reduced CD4^+^ T cell counts and downregulated antigen presentation pathway via MHC-II (57) in the injured *VEGFR3^iLECko^* iris tissue, compared with the injured *VEGFR3^fl/fl^* tissue (Figure 5H, lower panel, and Figure 5, I and J). Notably, the production of both Th1 (IFN-γ, IL-2, and TNF; ref. 58) and Th2 cytokines (IL-4, IL-5, and IL-13; refs. 59, 60) were greatly induced in the injured iris, while only TNF production was significantly inhibited upon *VEGFR3* deletion (Figure 5H and Supplemental Figure 5, A and B), indicating that the iridial neolymphatics may favor a Th1-type inflammatory response following CAI. The production of other major proinflammatory cytokines, such as IL-1β and IL-6, that were highly activated by CAI were not diminished in the injured *VEGFR3^iLECko^* iris tissue (Supplemental Figure 5B, lower panel). Interestingly, *VEGFR3^iLECko^* did not alter CD8^+^ T cell infiltration or its related GSEA pathways, such as antigen presentation pathway via MHC-I, CD8^+^ T cell activation, and T cell–mediated cytotoxicity, in the injured iris (Figure 5G, lower panel). Moreover, *VEGFR3^iLECko^* only slightly reduced FOXP3^+^ Treg counts in the injured iris tissue but did not significantly affect Treg-related pathways (Figure 5, I and J, and Supplemental Figure 5C, right panel).

With respect to B cell–mediated pathways, we found that gene sets for B cell activation and immunoglobin production were greatly upregulated in the iris after CAI. These were unaltered by *VEGFR3^iLECko^* (Supplemental Figure 6A). Similarly, neutrophil and Th17 pathways/cytokines (61) were largely induced in the injured iris but remained unchanged upon *VEGFR3* deletion (Supplemental Figure 6B). These data suggest that lymphangiogenic *VEGFR3* signaling is dispensable for B cell– and neutrophil-mediated immune pathways in the iris after CAI.

Collectively, our results suggest that the iridial neolymphatics contribute to phagocytosis, antigen presentation via MHC-II, and subsequent infiltration/activation of T cells, thereby promoting CAI-induced innate and adaptive immune responses.

Interestingly, GSEA also revealed significantly upregulated epithelial cell differentiation and morphogenesis pathways, accompanied by increased collagen biosynthesis, in the injured *VEGFR3^iLECko^* versus injured *VEGFR3^fl/fl^* iridial tissues (Supplemental Figure 6C). These data imply that inhibition of lymphangiogenesis may have indirectly improved healing of the pigmented/nonpigmented epithelium in the injured iris.

### VEGFR3 deletion alleviates CAI-induced thickening of the retina and the enlargement of cervical lymph nodes.

**Figure 6:**
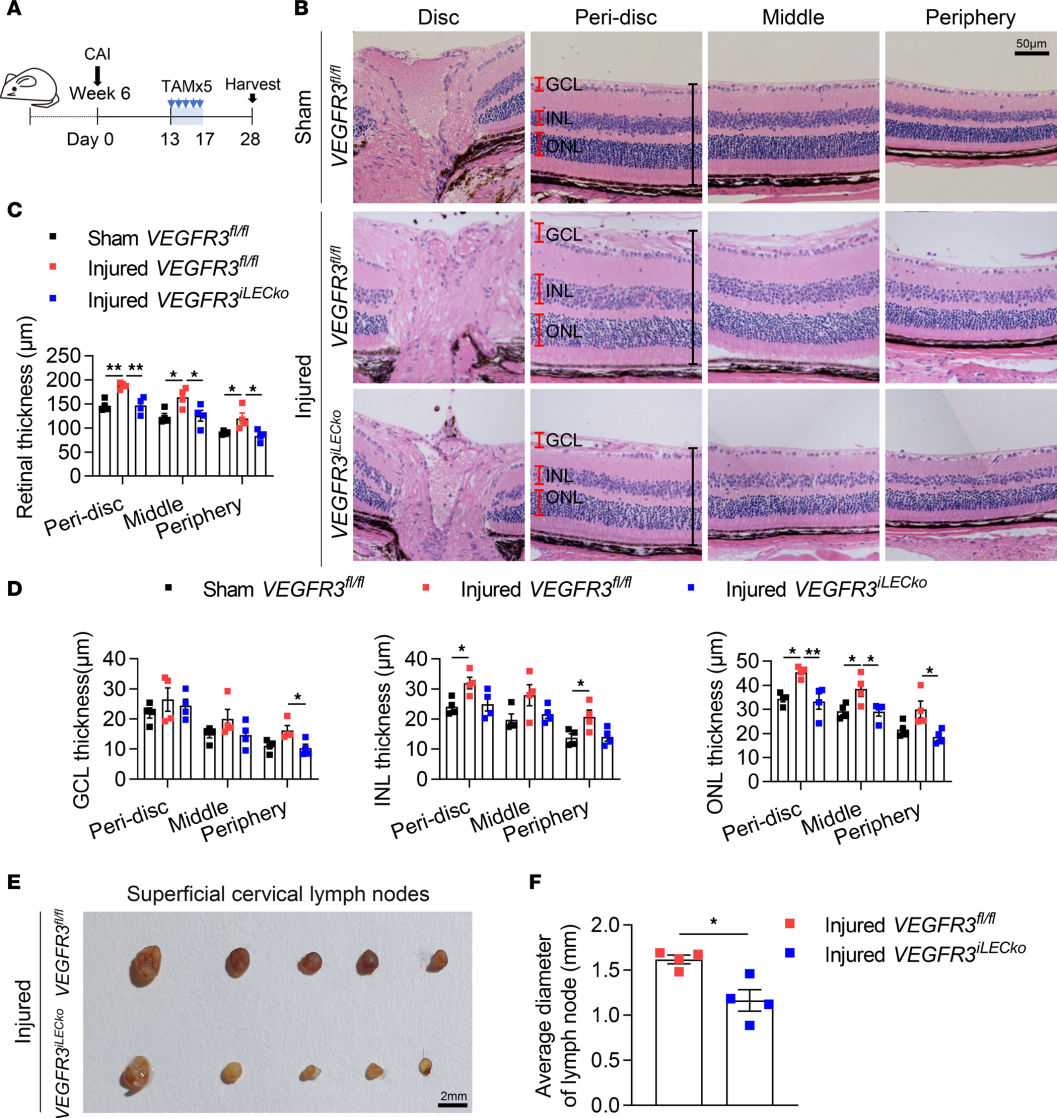
Inhibition of lymphangiogenesis alleviates CAI-induced retinal edema and enlargement of cervical lymph nodes. (**A**) Experimental timeline of TAM administration CAI in **B**–**F**. Mice received CAI or sham treatment at week 6, followed by TAM administered 5 times (80 mg/kg) i.p. between days 13 and 17 and analysis on day 28 after CAI. (**B**) Histological assessment of CAI-induced retina edema. Shown are representative images of the optic disc, peri-disc, middle, and peripheral regions in cross-sectioned retinas from *VEGFR3^iLECko^* and *VEGFR3^fl/fl^* mice with CAI or sham treatment. Scale bar: 50 μm. GCL, ganglion cell layer; INL, inner nuclear layer; ONL, outer nuclear layer. (**C**) Quantitation of thickness of the peri-disc, middle, and peripheral retinal regions in **B**. Data are shown as mean ± SEM. *n* = 4 mice per group. Each dot represents 1 mouse. *P < 0.05, **P < 0.01. One-way ANOVA with the Tukey post hoc test. (**D**) Quantitation of GCL, INL, and ONL thickness in the peri-disc, middle, and peripheral retinal regions in **B**. Data are shown as mean ± SEM. *n* = 4 mice per group. Each dot represents 1 mouse. *P < 0.05, **P < 0.01. One-way ANOVA with the Tukey post hoc test. (**E** and **F**) Images and the average diameter of cervical lymph nodes from *VEGFR3^iLECko^* and *VEGFR3^fl/fl^* mice with CAI or sham treatment. Scale bar: 2 mm. Data are shown as mean ± SEM. Each dot represents the average diameter of lymph nodes obtained from 1 mouse. *n* = 4 mice per group. *P < 0.05. Mann-Whitney *U* test.

Alkali injury eventually causes retinal damage due to ocular inflammation (40). We next investigated the functional influence of intraocular lymphangiogenesis on the retina by analyzing the retinal structure of *VEGFR3^iLECko^* and *VEGFR3^fl/fl^* mice with or without CAI (Figure 6A). We found that CAI clearly resulted in thickening of the ganglion cell layer (GCL), the inner nuclear layer (INL), and the outer nuclear layer (ONL) in *VEGFR3^fl/fl^* controls, indicating retinal injury by intraocular inflammation (Figure 6, B–D). This effect was significantly alleviated in CAI *VEGFR3^iLECko^* mice (Figure 6, B–D), indicating that intraocular lymphangiogenesis promoted ocular inflammation and injury in this context. Moreover, the size of cervical lymph nodes was reduced in the injured *VEGFR3^iLECko^* mice, compared with that in the injured *VEGFR3^fl/fl^* controls (Figure 6, E and F). Collectively, these data suggest that inhibition of pathological lymphangiogenesis may alleviate intraocular inflammation and the systemic adaptive immune response.

## Discussion

Previous studies have identified extraocular lymphatics in the conjunctiva, limbus, dura mater of the optic nerve, and lacrimal gland in the orbit and eye (62, 63), whereas a classic lymphatic system is not detectable intraocularly in mammals under normal conditions (30, 64–66). Here, we report, for the first time to our knowledge, that CAI can induce the formation of lymphatic vessels in the iris in a *VEGFR3*-dependent manner and that the neolymphatics contribute to the process of antigen presentation and the subsequent T cell–mediated immune response in the iris following injury.

Infection or inflammation has been frequently shown to induce lymphangiogenesis (12). At the early stage of inflammation, the activation of proinflammatory signaling pathways, such as NF-κB, could upregulate VEGFR3 expression in LECs to promote lymphangiogenesis (67). Inflammation also triggers the infiltration and activation of macrophages that can produce prolymphangiogenic factors such as VEGFC to further potentiate this pathological process (68). Historically, it has been widely acknowledged that prolymphangiogenic factors elicit LEC sprouting from existing lymphatics of the venous origin to form neolymphatics postnatally. However, recent studies have demonstrated the diversity and complexity of LEC progenitors under physiological and pathological conditions (2, 46, 48, 69, 70). For example, a substantial portion of LECs in cardiac and dermal lymphatics have nonvenous cell origins (2, 69). CD11b^+^ macrophages can also transdifferentiate into LECs and form lymphatic-like tubes after mouse corneal transplantation or suture (14). Similarly, TLR4 activation–dependent myeloid-to-lymphatic transition can give rise to LEC precursors and promote inflammatory lymphangiogenesis in peritonitis mice (71). Our data suggest that CAI-induced iridial neolymphatics originate from CDH5^+^Prox1^+^ LECs of existing lymphatics in, presumably, the limbus and that these vessels do not have a CX3CR1^+^ myeloid or PDGFRB^+^ mural cell origin.

The iridial neolymphatics appear to have a detrimental effect on the injured eye within the 4-week time window following CAI, in contrast to the physiological lymphatic network that is apparently beneficial to tissue homeostasis. The ocular environment is traditionally defined as an immune privilege site due to its immunological ignorance, induction of systemic tolerance, and regional immune suppression (31). According to the Medawar’s hypothesis, the alymphatic nature of intraocular tissues is necessary to shield antigens from the immune system (72). Previous studies have shown that the immunological surveillance populations in the healthy anterior uveal tract are mainly composed of resident macrophages and DCs that maintain homeostasis and participate in the response to pathological challenges (73, 74), and they fail to migrate to draining lymph nodes even with antigen or LPS stimulation (75). Our data reveal that CAI causes drastic inflammatory responses involving infiltration/activation of broad-spectrum immune cells, including systemic immune cells (such as T cells, B cells, and neutrophils) and resident cells (such as macrophages and DCs), in the injured tissue. Ablation of lymphangiogenesis via *VEGFR3* deletion only partially inhibited DC/macrophage migration, phagocytosis, antigen presentation via MHC-II, and infiltration/activation of pathogenic T cell populations, such as CD4^+^ Th cells, without significantly affecting B cell, neutrophil, or CD8^+^ T cell–mediated immunogenic pathways. Thus, CAI-induced lymphatics could be immature and inferior, as evidenced by the formation of zipper-like junctions in the terminal segments and the following plexus of the neolymphatic, but they can still transport antigens and immune cells by connecting the iridial regional microenvironment with the draining lymph nodes and the systemic lymphatic network and play a regulatory role in certain aspects of CAI-induced innate and adaptive immune responses (e.g., phagocytosis, antigen presentation, and T cell infiltration/activation), thereby impeding intraocular immune privilege. This, at least partially, resembles graft rejection diseases where lymphatic vessels serve as an immune reflex arc to facilitate the migration of antigen presenting cells to draining lymph nodes and to evoke the subsequent activation of pathogenic CD4^+^/CD8^+^ T cell subsets for graft rejection (24, 76–78). Such a pathogenic effect of neolymphatics was observed in cornea, liver, or kidney allograft rejection (79–81), and antilymphangiogenesis therapy can prolong graft survival (82, 83). Similarly, allergen-induced conjunctival or airway lymphangiogenesis can promote antigen presentation and the subsequent allergy-specific inflammatory cascade and clinical signs (27, 84). By contrast, lymphatic vessels are capable of ameliorating chronic inflammation and edema in the bacterial keratitis model (85), reinforcing the notion that lymphangiogenesis plays context-dependent roles in different disease and tissue settings. Furthermore, the lymphatic vasculature has not been well studied in the autoimmune conditions, but current evidence supports the idea that lymphatic dysfunction may contribute to the development of autoimmunity (86). While uveitis is a frequent complication of systemic autoimmune disorders (87), whether such a condition can induce lymphangiogenesis in the uveal tract requires further studies.

Of note, we found that intracameral injections PBS in the second week following CAI can alleviate neolymphatic formation and T cell accumulation in the iris (see PBS injection in Figure 3E and Figure 5E). We speculate that paracentesis per se may have diluted the proinflammatory cytokines and prolymphangiogenic factors in the anterior chamber, thereby attenuating regional inflammation and lymphangiogenesis. As such, surgical management could be a valid option to relieve intraocular inflammation at the early stage after injury.

Still, our findings were only based on mouse experiments in a relatively short period after injury. Whether injury can also induce lymphangiogenesis in the iris or other intraocular tissues in patients and, if it can, how this affects the ocular pathophysiology in the long-term remain to be investigated. Recent advancements in the development of state-of-the-art lymphangiography (88) may aid in establishing the pathways/patterns of lymphatic drainage in intraocular tissues and in guiding for possible diagnostic and interventional applications. Furthermore, a more comprehensive single-cell transcriptomic analysis may be a better approach to understanding the complex immunogenic pathways in the residential and circulating immune cells and their crosstalk with the neolymphatics in the context of CAI or other inflammatory eye injury diseases; this research is ongoing.

In summary, this work reveals injury-induced lymphangiogenesis in the iris and establishes its detrimental effect on intraocular inflammation. We suggest that the iridial neolymphatics promote phagocytosis and antigen presentation process and facilitate the subsequent T cell–mediated adaptive immune response, thereby deteriorating regional inflammation and retinal injury. Finally, we show that inhibition of VEGFR3-dependent lymphangiogenesis is beneficiary to the prevention of intraocular tissue damage following CAI, thus providing a therapeutic target for ocular inflammatory diseases.

## Methods

### Sex as a biological variable.

Our study examined male and female animals, and similar findings are reported for both sexes.

### Mice and inducible genetic experiments.

All mice were on a C57BL/6J background and were maintained in-house under specific pathogen–free (SPF) conditions. *VEGFR3^fl/fl^* (89), *Prox1-CreER^T2^* (50), and *CDH5-CreER^T2^* (51) mice were provided by K. Alitalo (University of Helsinki, Helsinki, Finland), T. Makinen, and R. Adams (both from Cancer Research UK London Research Institute, London). *CAG-tdTomato* (The Jackson Laboratory, 007909) (49) reporter mice were interbred with *Prox1-CreER^T2^*, *CDH5-CreER^T2^, PDGFRB-CreER^T2^* (The Jackson Laboratory, 029684) (52), or *CX3CR1-CreER^T2^* (The Jackson Laboratory, 021160) (90) mice. For genetic cell fate tracking, TAM (MilliporeSigma, T5648; 3 × 80 mg/kg body weight; dissolved in peanut oil) was administered i.p. to *CAG-tdTomato;Prox1-CreER^T2^, CAG-tdTomato;CDH5-CreER^T2^, CAG-tdTomato;PDGFRB-CreER^T2^*, or *CAG-tdTomato;CX3CR1-CreER^T2^* mice at postnatal week 4. LEC-specific *VEGFR3* depletion was induced in *VEGFR3^fl/fl^*;*Prox1CreER^T2^* mice by daily i.p. injections of TAM (80 mg/kg body weight) on days 13–17 after CAI (see below), and littermate *VEGFR3^fl/fl^* mice that received the same TAM administration were used as controls. In the indicated experiments, WT mice were randomly allocated to receive 3 intracameral injections of 150 ng recombinant human VEGFC-156S (R&D Systems, 752-VC) (2) or PBS on days 16–18 after CAI. Animals were included based on their proper age and genotype, and both male and female mice were used.

### CAI model.

The CAI model was established as described previously (40). Six-week-old mice were anesthetized with 50 mg/kg pentobarbital sodium and placed on a body warming pad, and topical anesthesia eye drop (proparacaine hydrochloride, Alcon, 1370) was applied to the cornea for 30 seconds. A 2 mm round filter paper infiltrated with 0.5 μL of 1 M sodium hydroxide solution (Sigma, S2770) was placed on the cornea for 60 seconds with no direct contact with the limbus. Thereafter, the injured cornea was gently rinsed with sterile saline for at least 60 seconds and covered by topical antibiotic gel (Ofloxacin Eye Ointment, Shenyang Xingqi Pharmaceutical Co.). PBS-operated mouse eyes were used as the sham controls. Slit-lamp observations and pharmacological procedures at later time points were performed as indicated.

### Immunofluorescence staining.

For whole-mount staining of the iris and cornea, mice under anesthesia were perfused with PBS and then 2% paraformaldehyde (PFA, Sigma, 158127). Enucleated eyeballs were then fixed in 4% PFA for another 30 minutes. The iris and cornea whole tissues were dissected, rinsed with PBS, and blocked with a solution containing 5% donkey serum (Solarbio, SL050), 2% BSA (MP Biomedicals, 2180728), and 1% Triton X-100 (Sigma, X100-500ML) for 1 hour, then incubated with the indicated primary antibodies (Supplemental Table 1) overnight in a cold room and with fluorescently labeled secondary antibodies for 1.5 hours at room temperature with three 15-minute PBS washes after each antibody incubation. Alternatively, frozen sectioned eyeball samples (20 μm in thickness) were processed and subjected to immunostaining using appropriate antibodies as indicated.

### Histology.

Eyeballs were collected, fixed with 4% PFA for 2 hours, and processed according to the standard paraffin embedding and sectioning procedure. H&E or Masson’s trichrome staining was then performed.

### Image data collection and quantitation.

All stitched or unstitched fluorescence microscopy images of the tissue whole mounts or sections were captured using a Nikon CS2 confocal microscope or a Tissue FAXS (TissueGnostics) imaging system. The density, diameter, sprouting branch counts, and vessel endpoint counts of all LYVE1^+^CD31^+^ or Prox1^+^ lymphatic vessels were measured in the merged whole-plane images of the corneal or iridial whole mounts using ImageJ software (NIH) (91). The percentage of tdTomato^+^ lymphatic area out of LYVE1^+^Prox1^+^ lymphatic area or the density of immune cells was measured in at least 3 fields of interest per mouse eye sample. Cervical lymph nodes (4–8 per mouse) were obtained, and their short-axis diameter was measured. Data were then averaged, and the values in individual mice were reported.

### RNA-Seq.

For bulk RNA-Seq analysis, the iris tissues were collected at week 10 from *VEGFR3^iLECko^* and *VEGFR3^fl/fl^* mice that received CAI or sham treatment at week 6. In brief, the anterior segment of the eyeball was dissected. After complete removal of the shell of the anterior segment (the cornea and limbus) and the lens and surrounding zonular fibers, a blunt dissection was performed at the root of the iris (anterior to the ciliary body) to isolate the iris from the ciliary body. To exclude any adjacent tissues attached on the iris, we carefully trimmed the outer edge (~1 mm) of the iris tissue. The remaining iris tissue (~3 mm in diameter) was used for RNA-Seq analyses. To obtain sufficient and eligible RNA, 3 mouse irises with identical genotypes were combined as 1 sample (3 samples in each group), and 3 sequencing experiments (3 × 3 mice in each group) were conducted. RNA quality was evaluated by using a Fragment Analyzer. RNA-Seq libraries were generated from 1 μg of qualified RNA using the Illumina TruSeq mRNA sample preparation kit (Illumina). Sequencing was performed on DNBSEQ with a read length of SE50 on the BGIseq500 platform (BGI Group). Filtered clean reads were aligned to the mouse genome using hierarchical indexing for spliced alignment of transcripts 2 (HISAT2, v2.0.4) and Bowtie2 (v2.2.5). After removal of rare genes (transcripts per million [TPM] < 2 in all groups), gene expression comparisons of DEGs (*P*_adj_ < 0.05) were conducted using DESeq2 (v1.4.5) with the following thresholding parameters: fold change > 1.5 and *P*_adj_ < 0.05 after Benjamini-Hochberg multiple testing corrections. We then confirmed that expression of ATP6V1C2 and CACNA1E, which are responsible for humor secretion and transportation in the ciliary body (92), were undetectable (TPM < 1) in all data sets, thereby ensuring that the iris tissue was not contaminated by the adjacent ciliary body tissue. Thereafter, a volcano plot and Venn diagram were generated, and KEGG and GO analyses were conducted based on the identified DEGs. GSEA of the RNA-Seq data was also performed, with *P*_adj_ < 0.05 (after Benjamini-Hochberg multiple testing corrections) to be considered statistically significant. Please refer to Supplemental Table 2 for the indicated GO terms.

### Statistics.

Data (except for the RNA-Seq data) were analyzed using Prism 9 (GraphPad Software). Statistical power analyses using the preliminary animal model data indicate that a sample size of at least 4 mice per group in the morphological tests for lymphatic coverage, width, sprouting branch counts, and endpoints is necessary to achieve a 0.8 power and α = 0.05. For cell count experiments, multiple measurements based on at least 3 fields of interest per mouse eye sample were averaged prior to statistical analysis. Scatterplots of individual data points with the mean ± SEM are shown. Comparisons between 2 groups were done by Mann-Whitney *U* test, and multiple-group comparisons were performed by 1-way ANOVA followed by the Tukey post hoc test (for data with distribution normality and equal variances) or Welch’s 1-way ANOVA followed by the Dunnett T3 post hoc test (for data with distribution normality and unequal variances). A *P* value less than 0.05 was considered statistically significant.

### Study approval.

All animal experiments were conducted in compliance with the requirements of the IACUC of Zhongshan Ophthalmic Center, Sun Yat-sen University, and of the Use of Animals in Ophthalmology and Vision Research.

### Data availability.

The raw data sets and matrix files from the RNA-Seq analysis are available in the NCBI GEO database (accession no. GSE240269). Values for all data points in graphs are reported in the Supporting Data Values file.

## Author contributions

ZL, KL, SS, SS, XC, XG, WW, KM, and RY performed the experiments. ZL, WW, LZ, WW, and WZ carried out data analysis. BL, FZ, and XL provided critical reagents or expertise. ZL, BL, XL, and FZ designed the experiments and wrote the manuscript.

## Supplementary Material

Supplemental data

Supporting data values

## Version 1 04/08/2024

Electronic publication

## References

[1] Petrova TV, Koh GY (2020). Biological functions of lymphatic vessels. Science.

[2] Klotz L (2015). Cardiac lymphatics are heterogeneous in origin and respond to injury. Nature.

[3] Biswas L (2023). Lymphatic vessels in bone support regeneration after injury. Cell.

[4] D’Alessio S (2014). VEGF-C-dependent stimulation of lymphatic function ameliorates experimental inflammatory bowel disease. J Clin Invest.

[5] Hsu M (2019). Neuroinflammation-induced lymphangiogenesis near the cribriform plate contributes to drainage of CNS-derived antigens and immune cells. Nat Commun.

[6] Huggenberger R (2011). An important role of lymphatic vessel activation in limiting acute inflammation. Blood.

[7] Song E (2020). VEGF-C-driven lymphatic drainage enables immunosurveillance of brain tumours. Nature.

[8] Baluk P (2009). TNF-alpha drives remodeling of blood vessels and lymphatics in sustained airway inflammation in mice. J Clin Invest.

[9] Baluk P (2013). Transgenic overexpression of interleukin-1β induces persistent lymphangiogenesis but not angiogenesis in mouse airways. Am J Pathol.

[10] Tan KW (2014). Inflammatory lymphangiogenesis: cellular mediators and functional implications. Angiogenesis.

[11] Kataru RP (2009). Critical role of CD11b+ macrophages and VEGF in inflammatory lymphangiogenesis, antigen clearance, and inflammation resolution. Blood.

[12] Kim H (2014). Inflammation-associated lymphangiogenesis: a double-edged sword?. J Clin Invest.

[13] Cursiefen C (2014). VEGF-A stimulates lymphangiogenesis and hemangiogenesis in inflammatory neovascularization via macrophage recruitment. J Clin Invest.

[14] Maruyama K (2005). Inflammation-induced lymphangiogenesis in the cornea arises from CD11b-positive macrophages. J Clin Invest.

[15] Kerjaschki D (2006). Lymphatic endothelial progenitor cells contribute to de novo lymphangiogenesis in human renal transplants. Nat Med.

[16] Lee JY (2010). Podoplanin-expressing cells derived from bone marrow play a crucial role in postnatal lymphatic neovascularization. Circulation.

[17] Oliver G (2010). The lymphatic vasculature in the 21^st^ century: novel functional roles in homeostasis and disease. Cell.

[18] Brakenhielm E, Alitalo K (2019). Cardiac lymphatics in health and disease. Nat Rev Cardiol.

[19] Vieira JM (2018). The cardiac lymphatic system stimulates resolution of inflammation following myocardial infarction. J Clin Invest.

[20] Vuorio T (2014). Lymphatic vessel insufficiency in hypercholesterolemic mice alters lipoprotein levels and promotes atherogenesis. Arterioscler Thromb Vasc Biol.

[21] Hsu SJ (2021). Enhanced meningeal lymphatic drainage ameliorates neuroinflammation and hepatic encephalopathy in cirrhotic rats. Gastroenterology.

[22] Da MS (2018). Functional aspects of meningeal lymphatics in ageing and Alzheimer’s disease. Nature.

[23] Louveau A (2018). CNS lymphatic drainage and neuroinflammation are regulated by meningeal lymphatic vasculature. Nat Neurosci.

[24] Dietrich T (2010). Cutting edge: lymphatic vessels, not blood vessels, primarily mediate immune rejections after transplantation. J Immunol.

[25] Edwards LA (2018). Chronic rejection of cardiac allografts is associated with increased lymphatic flow and cellular trafficking. Circulation.

[26] Donnan MD (2021). The lymphatics in kidney health and disease. Nat Rev Nephrol.

[27] Lou B (2022). Alleviating experimental allergic eye disease by inhibiting pro-lymphangiogenic VEGFR3 signal. Ocul Surf.

[28] Medoff BD (2008). T cell trafficking in allergic asthma: the ins and outs. Annu Rev Immunol.

[29] Engelhardt B (2017). The movers and shapers in immune privilege of the CNS. Nat Immunol.

[30] Clahsen T (2023). The novel role of lymphatic vessels in the pathogenesis of ocular diseases. Prog Retin Eye Res.

[31] Streilein JW (2003). Ocular immune privilege: therapeutic opportunities from an experiment of nature. Nat Rev Immunol.

[32] Lou B (2022). A single-cell transcriptomic atlas of the human ciliary body. Cell Mol Life Sci.

[33] Bock F (2016). Identification of novel endogenous anti(lymph)angiogenic factors in the aqueous humor. Invest Ophthalmol Vis Sci.

[34] Bock F (2013). Novel anti(lymph)angiogenic treatment strategies for corneal and ocular surface diseases. Prog Retin Eye Res.

[35] Shi W (2011). Features of corneal neovascularization and lymphangiogenesis induced by different etiological factors in mice. Graefes Arch Clin Exp Ophthalmol.

[36] Dua HS (2020). Chemical eye injury: pathophysiology, assessment and management. Eye (Lond).

[37] Choi H (2017). Comprehensive modeling of corneal alkali injury in the rat eye. Curr Eye Res.

[38] Yan H (2010). Lymphatic vessels correlate closely with inflammation index in alkali burned cornea. Curr Eye Res.

[39] Aketa N (2017). Iris damage is associated with elevated cytokine levels in aqueous humor. Invest Ophthalmol Vis Sci.

[40] Paschalis EI (2017). Mechanisms of retinal damage after ocular alkali burns. Am J Pathol.

[41] Quesada JM (2020). Ocular chemical burns in the workplace: epidemiological characteristics. Burns.

[42] Wigle JT, Oliver G (1999). Prox1 function is required for the development of the murine lymphatic system. Cell.

[43] Dumont DJ (1998). Cardiovascular failure in mouse embryos deficient in VEGF receptor-3. Science.

[44] Zhang F (2020). Lymphatic endothelial cell junctions: molecular regulation in physiology and diseases. Front Physiol.

[45] Liao S, Von der Weid PY (2014). Inflammation-induced lymphangiogenesis and lymphatic dysfunction. Angiogenesis.

[46] Srinivasan RS (2007). Lineage tracing demonstrates the venous origin of the mammalian lymphatic vasculature. Genes Dev.

[47] Pichol-Thievend C (2018). A blood capillary plexus-derived population of progenitor cells contributes to genesis of the dermal lymphatic vasculature during embryonic development. Development.

[48] Ulvmar MH (2016). Pdgfrb-Cre targets lymphatic endothelial cells of both venous and non-venous origins. Genesis.

[49] Madisen L (2010). A robust and high-throughput Cre reporting and characterization system for the whole mouse brain. Nat Neurosci.

[50] Bazigou E (2011). Genes regulating lymphangiogenesis control venous valve formation and maintenance in mice. J Clin Invest.

[51] Wang Y (2010). Ephrin-B2 controls VEGF-induced angiogenesis and lymphangiogenesis. Nature.

[52] Gerl K (2015). Inducible glomerular erythropoietin production in the adult kidney. Kidney Int.

[53] Zhao XF (2019). Targeting microglia using Cx3cr1-cre lines: revisiting the specificity. eNeuro.

[54] Visuri MT, H et al (2015). VEGF-C and VEGF-C156S in the pro-lymphangiogenic growth factor therapy of lymphedema: a large animal study. Angiogenesis.

[55] Dick SA (2019). Self-renewing resident cardiac macrophages limit adverse remodeling following myocardial infarction. Nat Immunol.

[56] Gordon S, Martinez FO (2010). Alternative activation of macrophages: mechanism and functions. Immunity.

[57] Kasuga Y (2023). FBXO11 constitutes a major negative regulator of MHC class II through ubiquitin-dependent proteasomal degradation of CIITA. Proc Natl Acad Sci U S A.

[58] Voskuhl RR (1993). T helper 1 (Th1) functional phenotype of human myelin basic protein-specific T lymphocytes. Autoimmunity.

[59] Seder RA (1992). The presence of interleukin 4 during in vitro priming determines the lymphokine-producing potential of CD4+ T cells from T cell receptor transgenic mice. J Exp Med.

[60] Saravia J (2019). Helper T cell differentiation. Cell Mol Immunol.

[61] Wilson AS (2022). Neutrophil extracellular traps and their histones promote Th17 cell differentiation directly via TLR2. Nat Commun.

[62] Gausas RE (1999). Identification of human orbital lymphatics. Ophthalmic Plast Reconstr Surg.

[63] Wu Y (2020). Organogenesis and distribution of the ocular lymphatic vessels in the anterior eye. JCI Insight.

[64] Schrodl F (2015). Lymphatic markers in the adult human choroid. Invest Ophthalmol Vis Sci.

[65] Kaser-Eichberger A (2015). Topography of lymphatic markers in human iris and ciliary body. Invest Ophthalmol Vis Sci.

[66] Schroedl F (2014). Consensus statement on the immunohistochemical detection of ocular lymphatic vessels. Invest Ophthalmol Vis Sci.

[67] Flister MJ (2010). Inflammation induces lymphangiogenesis through up-regulation of VEGFR-3 mediated by NF-kappaB and Prox1. Blood.

[68] Glinton KE (2022). Macrophage-produced VEGFC is induced by efferocytosis to ameliorate cardiac injury and inflammation. J Clin Invest.

[69] Martinez-Corral I (2015). Nonvenous origin of dermal lymphatic vasculature. Circ Res.

[70] Antila S (2017). Development and plasticity of meningeal lymphatic vessels. J Exp Med.

[71] Volk-Draper LD (2017). Lymphatic endothelial progenitors originate from plastic myeloid cells activated by toll-like receptor-4. PLoS One.

[72] Medawar PB (1948). Immunity to homologous grafted skin; the fate of skin homografts transplanted to the brain, to subcutaneous tissue, and to the anterior chamber of the eye. Br J Exp Pathol.

[73] Chen L (2003). Macrophages and MHC class II positive dendritiform cells in the iris and choroid of the pig. Curr Eye Res.

[74] Lin W (2019). The role of ocular dendritic cells in uveitis. Immunol Lett.

[75] Dullforce PA (2004). APCs in the anterior uveal tract do not migrate to draining lymph nodes. J Immunol.

[76] Bachmann B (2010). Corneal neovascularization as a risk factor for graft failure and rejection after keratoplasty: an evidence-based meta-analysis. Ophthalmology.

[77] Pishesha N (2022). A guide to antigen processing and presentation. Nat Rev Immunol.

[78] Duneton C (2022). Activation and regulation of alloreactive T cell immunity in solid organ transplantation. Nat Rev Nephrol.

[79] Kerjaschki D (2004). Lymphatic neoangiogenesis in human kidney transplants is associated with immunologically active lymphocytic infiltrates. J Am Soc Nephrol.

[80] Dashkevich A (2010). Lymph angiogenesis after lung transplantation and relation to acute organ rejection in humans. Ann Thorac Surg.

[81] Cursiefen C (2003). Corneal lymphangiogenesis: evidence, mechanisms, and implications for corneal transplant immunology. Cornea.

[82] Nykanen AI (2010). Targeting lymphatic vessel activation and CCL21 production by vascular endothelial growth factor receptor-3 inhibition has novel immunomodulatory and antiarteriosclerotic effects in cardiac allografts. Circulation.

[83] Yin N (2011). Lymphangiogenesis is required for pancreatic islet inflammation and diabetes. PLoS One.

[84] Maisel K (2001). Pro-lymphangiogenic VEGFR-3 signaling modulates memory T cell responses in allergic airway inflammation. Mucosal Immunol.

[85] Narimatsu A (2019). Corneal lymphangiogenesis ameliorates corneal inflammation and edema in late stage of bacterial keratitis. Sci Rep.

[86] Ambler W (2022). Advances in understanding and examining lymphatic function: relevance for understanding autoimmunity. Curr Opin Rheumatol.

[87] Egwuagu CE (2021). Uveitis: molecular pathogenesis and emerging therapies. Front Immunol.

[88] Schwartz FR (2021). Lymphatic imaging: current noninvasive and invasive techniques. Semin Intervent Radiol.

[89] Haiko P (2008). Deletion of vascular endothelial growth factor C (VEGF-C) and VEGF-D is not equivalent to VEGF receptor 3 deletion in mouse embryos. Mol Cell Biol.

[90] He C (2021). A specific RIP3^+^ subpopulation of microglia promotes retinopathy through a hypoxia-triggered necroptotic mechanism. Proc Natl Acad Sci U S A.

[91] Buttner C (2019). Tyrosinase is a novel endogenous regulator of developmental and inflammatory lymphangiogenesis. Am J Pathol.

[92] Van Zyl T (2022). Cell atlas of the human ocular anterior segment: Tissue-specific and shared cell types. Proc Natl Acad Sci U S A.

