# Supplementary material for: Alkali injury–induced pathological lymphangiogenesis in the iris facilitates the infiltration of T cells and ocular inflammation
Source: JCI Insight. 2024 Apr 8;9(7):e175479. doi: 10.1172/jci.insight.175479 (PMC11128208; doi:10.1172/jci.insight.175479)
Supplement: Supplemental data [file jciinsight-9-175479-s113.pdf]

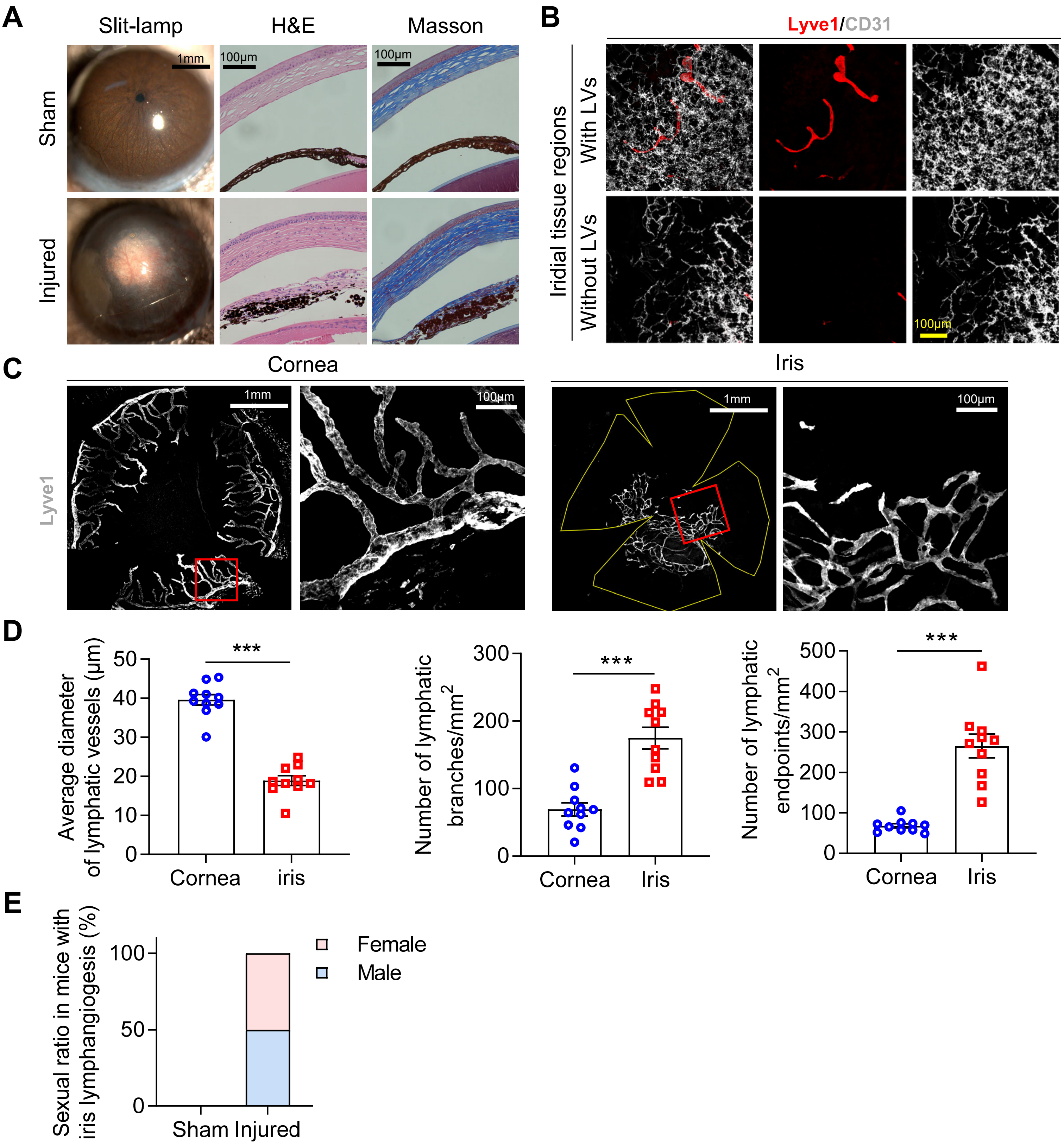

Supplementary Figure 1. Pathologic changes after corneal alkali injury (CAI).

A. Slit lamp images and histological analyses of wildtype mouse eyes with CAI or sham treatment. The CAI or sham treatment was done by placing a 2-mm round filter paper infiltrated with 0.5 μl of 1 M sodium hydroxide solution (CAI) or PBS (sham) on the cornea for 60 s. Note evident dilation and collagen deposition of the cornea and iris tissues in CAI-treated mouse group, as revealed by H&E and Masson staining of the cross-sectioned anterior segment. Scale bars: 1 mm and 100 μm.

B. Immunostaining for Lyve1 and CD31 of CAI-treated wildtype mouse iris tissue whole-mounts. Note prominent neovascularization in the injured iridial tissue regions, regardless of the presence or absence of lymphatic vessels (LVs). Scale bar: 100 μm.

C. Representative images for Lyve1 staining of cornea and iris tissue whole-mounts from wildtype mice that received CAI treatment at week 4, followed by analysis at week 10. Scale bars: 1 mm and 100 μm.

D. Quantifications of the diameter, branches and terminal endpoints of corneal/limbal and iridial lymphatics in **C**. Data are mean±SEM. Each dot represents one mouse. n=10 mice per group. *$p<0.05$, ***$p<0.001$. Mann-Whitney *U* test.

E. Sexual ratio of mice with iridial lymphangiogenesis at day 28 after CAI. n=10 mice (5 male and 5 female) per group.

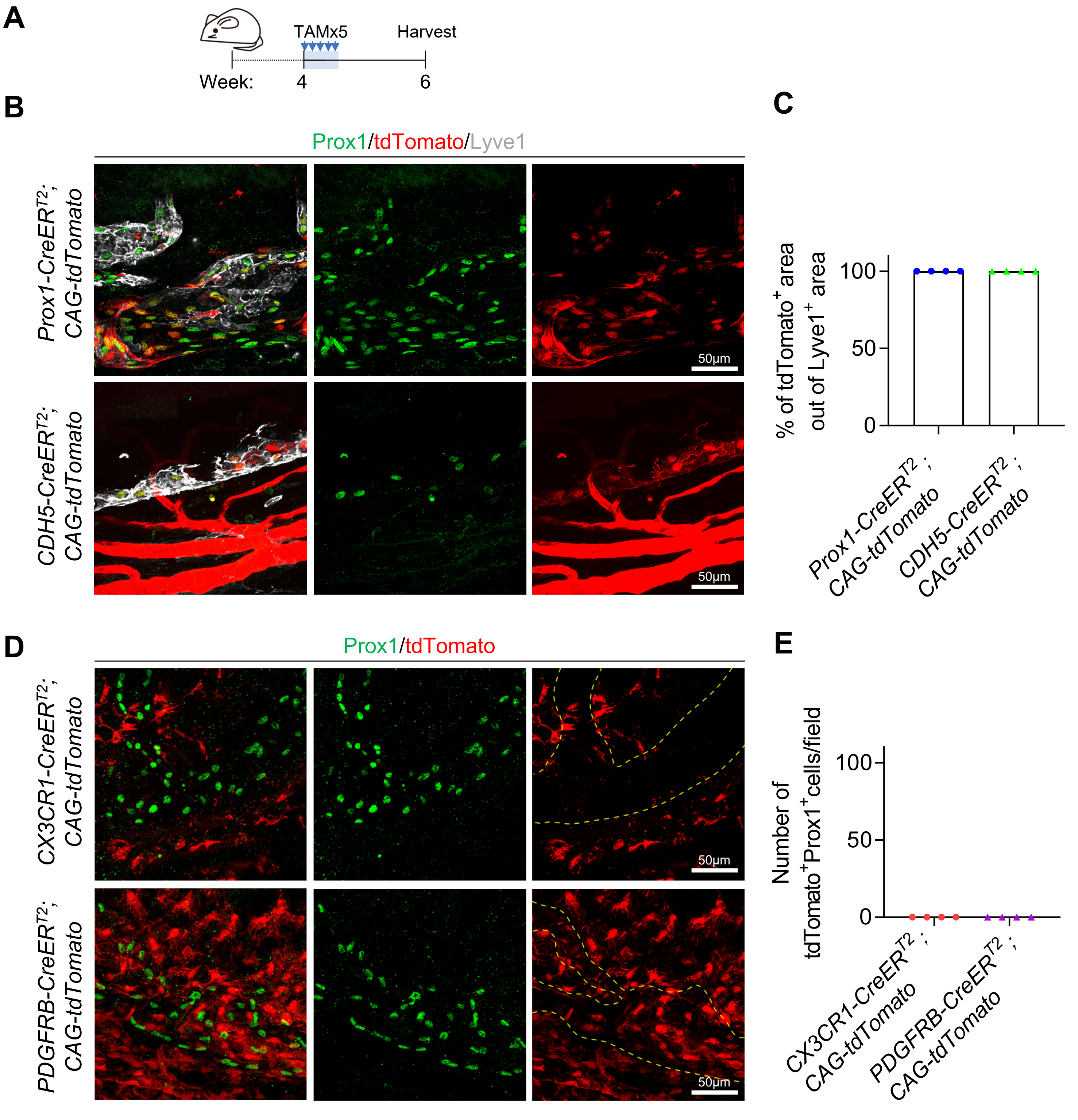

Supplementary Figure 2. Verification of lymphatic lineage tracing mice.

A. Experimental timeline of tamoxifen (TAM) administration for lineage tracing in the indicated tdTomato-reporter mouse lines (see **Figure 2**) in **B-E**. Arrows indicate 5XTAM (80 mg/kg) intraperitoneal (ip) injections in week 4, followed by analysis at week 6.

B. Representative tdTamoto, Prox1 and Lyve1 immunostaining images of the cornea tissue whole-mounts from *Prox1-CreER^{T2};CAG-tdTomato* and *CDH5-CreER^{T2};CAG-tdTomato*. All mice received 5XTAM ip injections in week 4 and were analyzed at week 6. Note overlapping of tdTomato-labeled area

with Prox1/Lyve1-labeled lymphatic areas in *Prox1-CreER^{T2};CAG-tdTomato*, *CDH5-CreER^{T2};CAG-tdTomato* mouse lines, confirming faithful labeling of LECs via *Prox1-CreER^{T2}* and *CDH5-CreER^{T2}*-driven recombination expression of tdTomato in existing lymphatics. Scale bar: 50 μm.

C. Quantification of the ratio of tdTomato-labeled area out of Prox1/Lyve1-labeled area in **B**. Data are mean±SEM. n=4 mice per group. Each dot represents one mouse.

D. Representative tdTomato and Prox1 immunostaining images of the cornea tissue whole-mounts from *PDGFRB-CreER^{T2};CAG-tdTomato* and *CX3CR1-CreER^{T2};CAG-tdTomato* mice. All mice received 5XTAM ip injections in week 4 and were analyzed at week 6. Note no double labeling tdTomato and Prox1 in *PDGFRB-CreER^{T2};CAG-tdTomato* and *CX3CR1-CreER^{T2};CAG-tdTomato* mouse lines. Scale bar: 50 μm.

E. Quantification of number of tdTomato and Prox1 double positive cells in **D**. Data are mean±SEM. n=4 mice per group. Each dot represents one mouse.

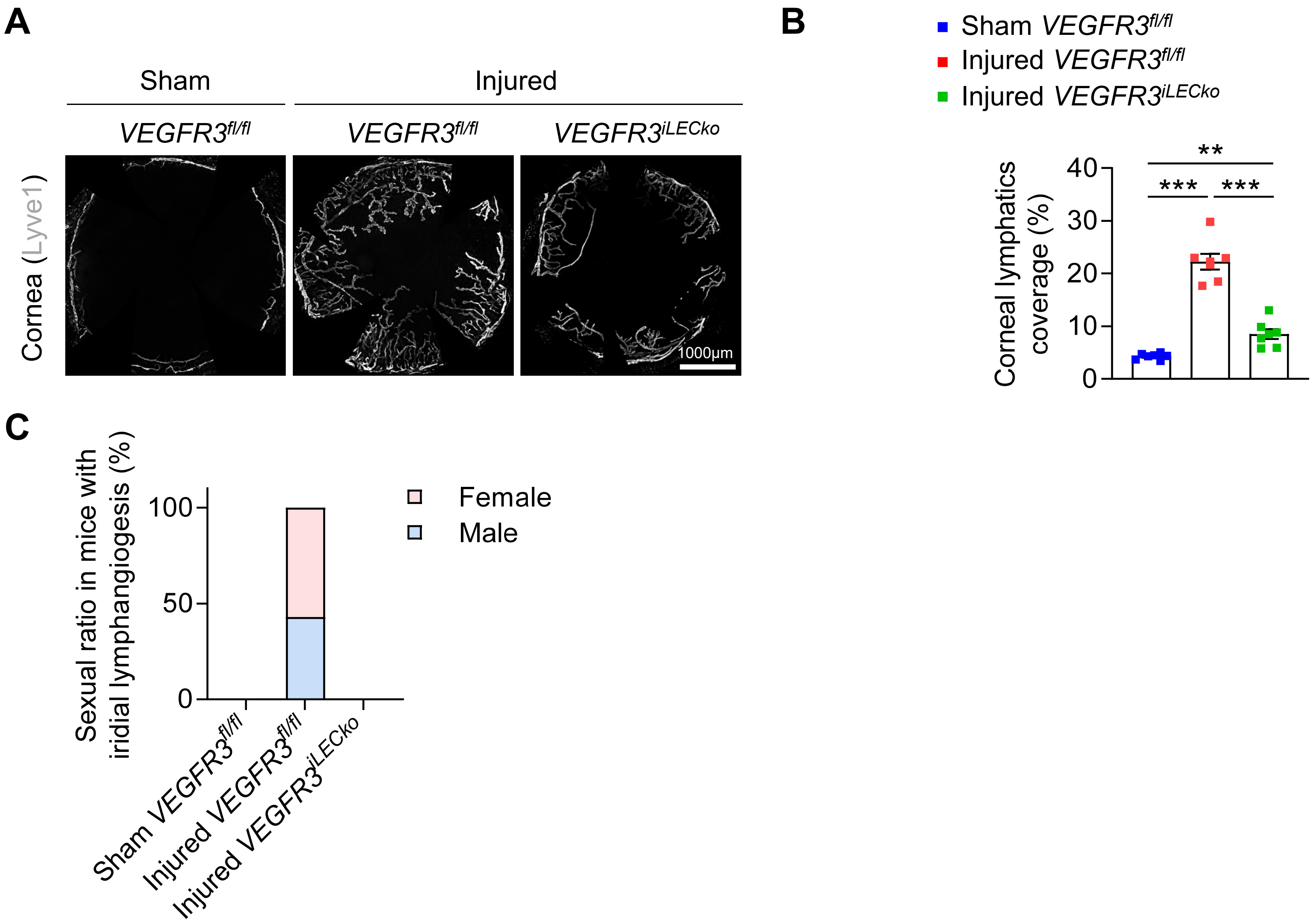

Supplementary Figure 3. Inhibition of CAI-induced corneal lymphangiogenesis in *VEGFR3^{iLECko}* mice.

A. Representative Lyve1 staining images of the cornea tissue whole-mounts from *VEGFR3$^{fl/fl}$* and *VEGFR3^{iLECko}* mice with CAI or sham treatment. Note that CAI induces drastic lymphangiogenesis in *VEGFR3$^{fl/fl}$* mice that is greatly inhibited in *VEGFR3^{iLECko}* mice. Scale bar: 1000 μm.

B. Quantification of corneal lymphatic coverage in mice in **A**. Data are mean±SEM. n=7 mice per group. Each dot represents one mouse. ** p<0.01. *** p<0.001. Welch's one-way ANOVA followed by the Dunnett T3 post hoc test.

C. Sexual ratio of mice shown in **Figure 3B-C**. n=7 mice (3 male+4 female) per group.

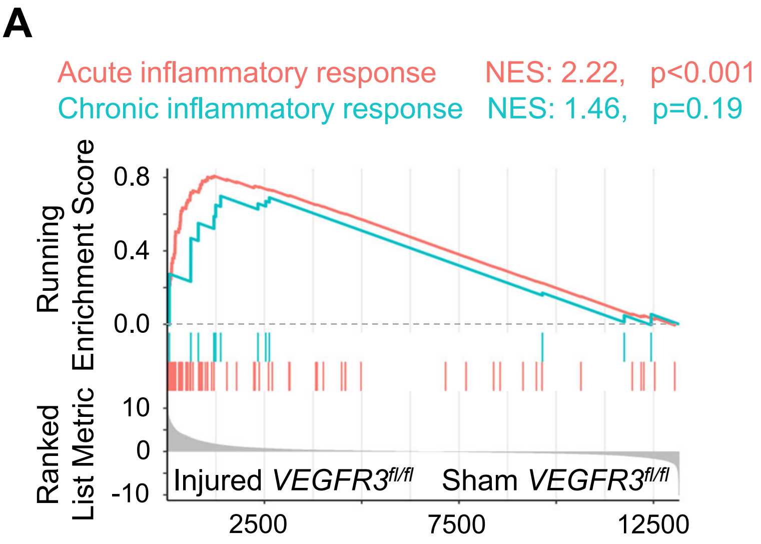

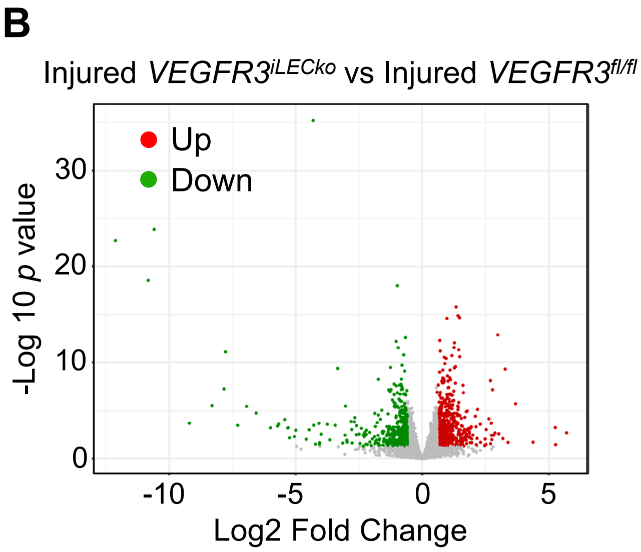

Supplementary Figure 4. RNA-seq analysis of inflammatory pathways in the iris following CAI and *VEGFR3* depletion.

A. GSEA showing expression pattern of acute and chronic inflammatory pathways following CAI. Adj.p value<0.05 (after Benjamini-Hochberg's multiple testing corrections) is considered statistically significant. NES: normalized enrichment score.

B. Volcano plot showing 259 upregulated differentially expressed genes (DEGs) and 222 downregulated DEGs in the Injured *VEGFR3*^{iLECko} vs. Injured *VEGFR3*$^{fl/fl}$ mouse iris tissues.

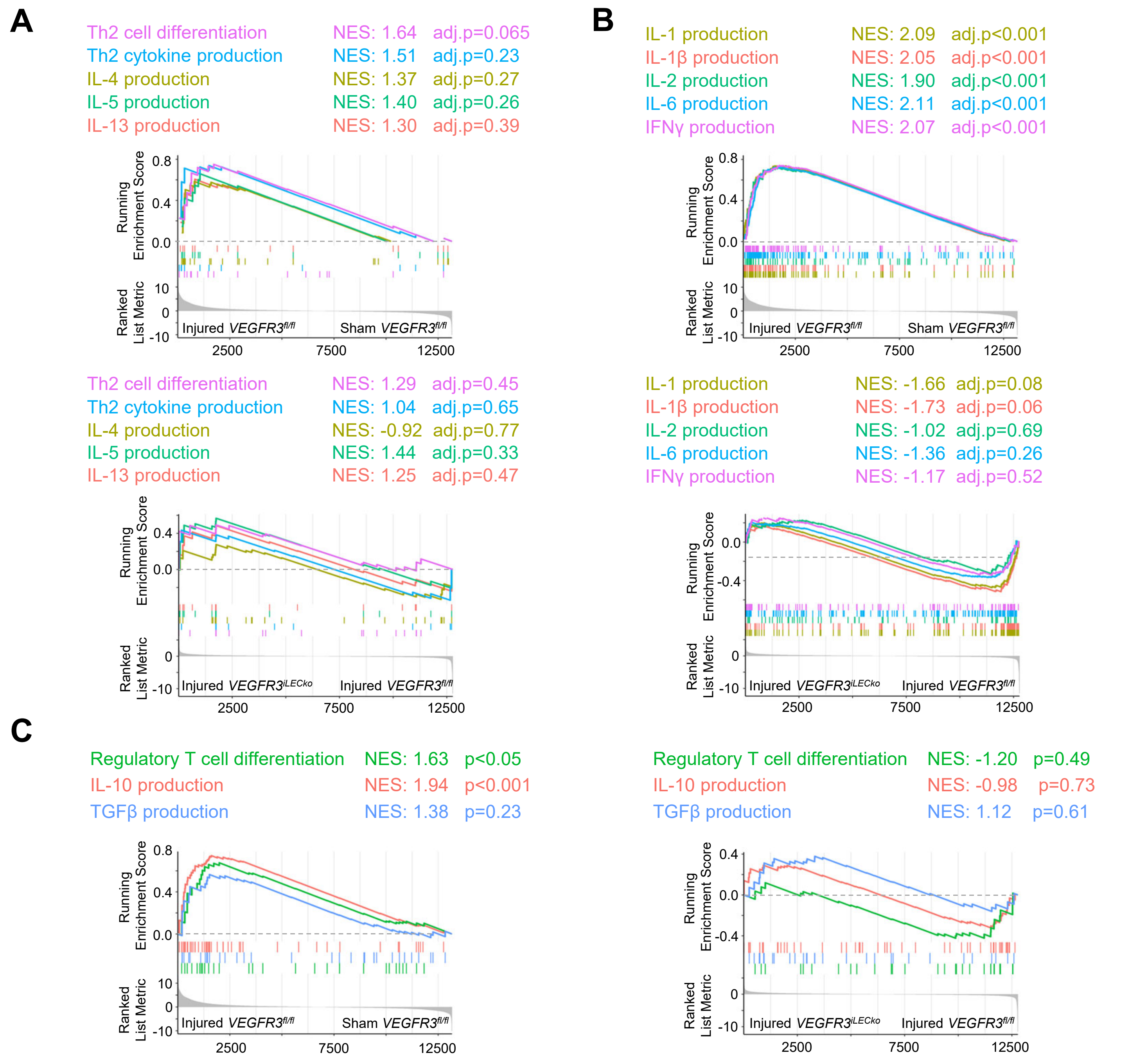

Supplementary Figure 5. GSEA showing expression profile of gene sets responsible for Th2-related pathway (**A**), proinflammatory cytokine production (**B**), regulatory T cell-related pathway (**C**) and in the indicated mouse iris tissues with or without CAI treatment. Adj.p value<0.05 (after Benjamini-Hochberg's multiple testing corrections) is considered statistically significant. NES: normalized enrichment score.

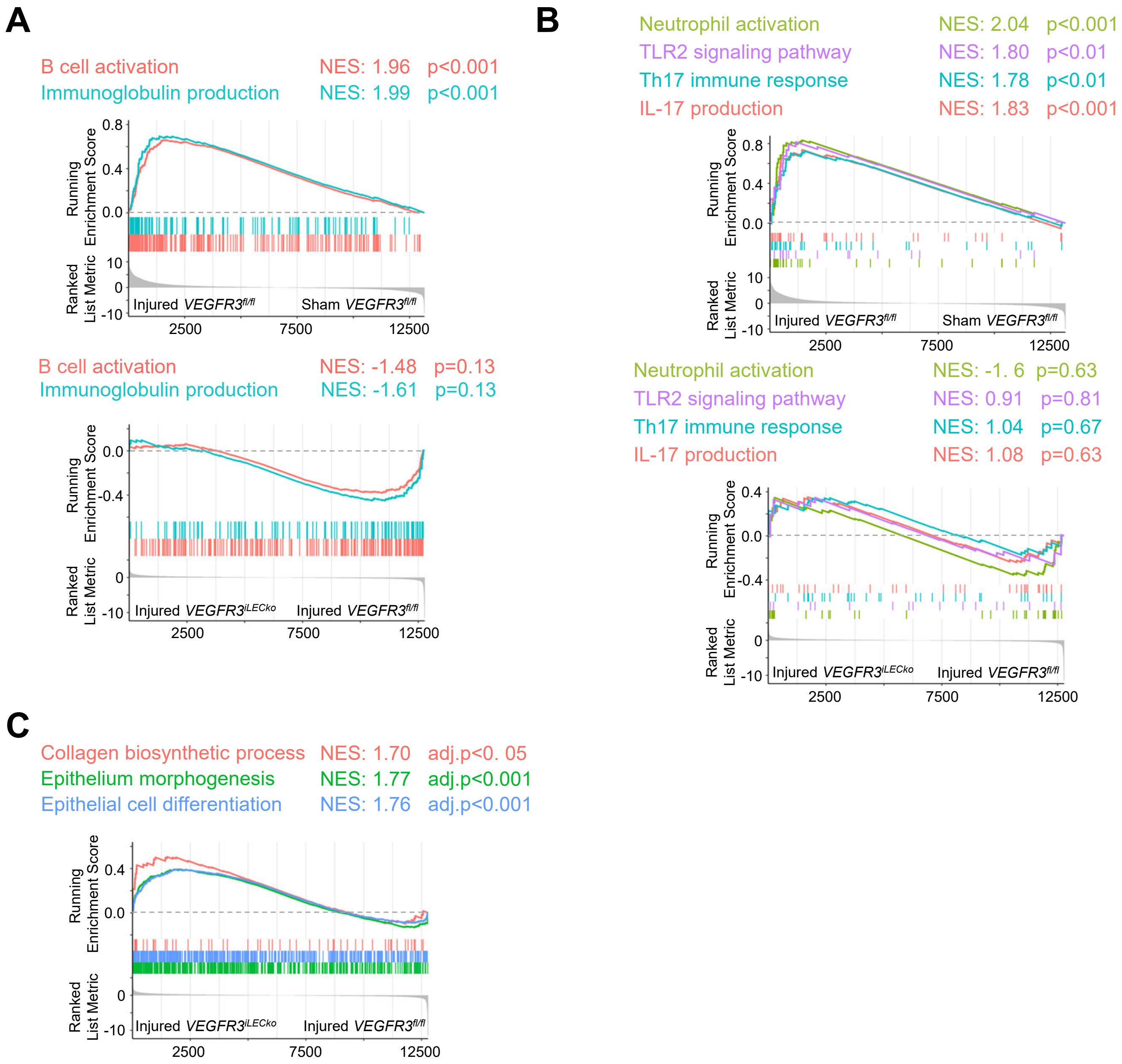

Supplementary Figure 6. GSEA showing expression profile of gene sets responsible for B-cell and Neutrophil-Th17-related pathways (**A-B**) and collagen synthesis and epithelium morphogenesis/differentiation (**C**) in the indicated mouse iris tissues with or without CAI treatment. Adj.p value<0.05 (after Benjamini-Hochberg's multiple testing corrections) is considered statistically significant. NES: normalized enrichment score.
